# The Continuum of Thyroid Disorders Related to Immune Checkpoint Inhibitors: Still Many Pending Queries

**DOI:** 10.3390/cancers13215277

**Published:** 2021-10-21

**Authors:** Maria V. Deligiorgi, Sofia Sagredou, Lampros Vakkas, Dimitrios T. Trafalis

**Affiliations:** Department of Pharmacology—Clinical Pharmacology Unit, Faculty of Medicine, National and Kapodistrian University of Athens, Building 16, 1st Floor, 75 Mikras Asias Str., Goudi, 11527 Athens, Greece; ssagredou@med.uoa.gr (S.S.); smd1700343@uoa.gr (L.V.); dtrafal@med.uoa.gr (D.T.T.)

**Keywords:** anti-CTLA-4 monoclonal antibodies, anti-PD-1 monoclonal antibodies, anti-PD-L1, monoclonal antibodies, immune checkpoint inhibitors, immune-related adverse events, hypothyroidism, hyperthyroidism, thyrotoxicosis, Graves’ disease, thyroid autoantibodies

## Abstract

**Simple Summary:**

To capitalize on the revolutionary anticancer efficacy of immune checkpoint inhibitors (ICPi) in an expanding list of tumors, including hematological malignancies, it is mandatory to mitigate the immune-related (ir) adverse events, among which prevail the ir thyroid disorders. Currently, most available data on ir thyroid disorders are derived from solid tumors. Our review provides a comprehensive and updated overview of ir thyroid disorders, dissecting several intriguing issues, namely: (i) the elusive biological background, (ii) the epidemiological profile, (iii) the clinical spectrum, (iv) the diagnostic and therapeutic algorithms, (v) the predictive value of antithyroid antibodies for the development of ir thyroid disorders, and (vi) the prognostic significance. To contribute to the decision-making, this review provides an on-hand presentation of the latest reviews, meta-analyses, and pharmacovigilance studies addressing ir thyroid disorders, as well as of the most widely applied treatment guidelines. The current challenges and future perspectives, as regards a tailored approach to ir thyroid disorders, are critically discussed.

**Abstract:**

Background: Until more data are available to shed light on the thyroid disorders related to immune checkpoint inhibitors (ICPi) implemented for the treatment of hematological malignancies, the decision-making is guided by pertinent data derived mostly from solid tumors. Methods: The present review provides a comprehensive and updated overview of the thyroid disorders related to ICPi, namely to inhibitors of cytotoxic T-lymphocyte antigen 4 (CTLA-4), programmed cell death (PD) 1 (PD-1), and the ligand of the latter (PD-L1). Results: With the increasing recognition of ir thyroid disorders, many outstanding issues have emerged. Ir thyroid disorders are reminiscent of, but not identical to, thyroid autoimmunity. Interclass and intraclass ICPi differences regarding thyroid immunotoxicity await interpretation. The available data concerning the predictive value of thyroid autoantibodies for the development of ir thyroid disorders are inconclusive. Mounting data indicate an association of ir thyroid disorders with ICPi efficacy, but a causative link is still lacking. The path forward is a tailored approach, entailing: (i) the validation of tumor-specific, patient-specific, and ICPi-specific predictive factors; (ii) appropriate patient selection; (iii) the uncoupling of antitumor immunity from immunotoxicity; (iv) a multidisciplinary initiative; and (v) global registry strategies. Conclusions: Untangling and harnessing the interrelationship of immuno-oncology with endocrinology underlying the ir thyroid disorders will yield the optimal patient care.

## 1. Introduction

The advent of the last decade witnessed a turning point in cancer therapeutics, signified by the incorporation of the immune checkpoint inhibitors (ICPi) in the oncologists’ arsenal [1]. Cancer cells coopt the immune checkpoints—inhibitory immune regulators credited with an assurance of immune tolerance—to escape from immune surveillance. A blockade of landmark immune checkpoints expressed on immune and cancer cells, namely the cytotoxic T-lymphocyte antigen 4 (CTLA-4), programmed cell death (PD) 1 (PD-1), and the ligand of the latter (PD-L1), harnesses the immune system to attack cancer cells [2,3]. The anti-CTLA-4 monoclonal antibodies (mAbs) reinvigorate T-cell activation in secondary lymphoid tissues and the intratumoral immune responses, while the anti-PD-1 mAbs preclude or delay T-cell exhaustion, thereby unleashing effector T-cell responses in tissues and tumors [3]. The approval of ipilimumab (anti-CTLA-4 monoclonal antibody) by the US Food and Drug Administration (FDA) for the treatment of metastatic melanoma in 2011 [4] paved the way for the approval of six, to date, additional ICPi—three anti-PD-1 (nivolumab, pembrolizumab, and cemiplimab) and three anti-PD-L1 (atezolizumab, durvalumab, avelumab) mAbs [5]—to revolutionize the treatment of non-small cell lung cancer (NSCLC), melanoma, renal cell carcinoma, head and neck squamous cell carcinoma (SCC), Merkel cell carcinoma, breast cancer (triple-negative), urothelial cancer, and squamous cell skin cancer. Such a milestone is inevitably interrelated with an ever-increasing constellation of immune-related (ir) adverse event(s) (irAE(s)), affecting every system. 

The miraculous therapeutic efficacy of ICPi in treating solid tumors inspired scientists to pursue the implementation of ICPi in the treatment of hematological malignancies. Several milestones as regards the efficacy of a PD-1 blockade in Hodgkin’s lymphoma (HL) led to the FDA approval of nivolumab (in 2016) and pembrolizumab (in 2017) for relapsed or refractory HL after the failure of multiple lines of therapy. The investigation of various ICPi in numerous hematological malignancies is currently ongoing, indicating that ICPi are efficient in certain hematological malignancies [2,3]. Due to the paucity of data on ir thyroid disorders in the setting of hematological malignancies, the decision-making is guided by relevant data derived mostly from solid tumors.

Real-world data leveraging the US FDA Adverse Event Reporting System (FAERS) and the WHO Vigibase (the largest worldwide databases collecting spontaneous reports) designate endocrine irAEs as the second-most often reported irAE from April 2011 until April 2015 [4] and the most common until February 2020 [5]. Ir thyroid disorders have emerged as the most frequently reported endocrine irAE [5,6]. Ir thyroid disorders are more strongly associated with anti-PD-1 mAbs, while a combination of ipilimumab with nivolumab is associated with a higher risk of thyroid disorders than expected with either agent as a monotherapy [7].

The spectrum of ir thyroid disorders is wide, comprising hypothyroidism, hyperthyroidism (mainly Graves’ disease), and destructive thyroiditis [6]. Ir destructive thyroiditis shows a typical triphasic course: thyrotoxicosis (i.e., status of increased serum T4 and T3 levels) due to destruction of the thyroid gland and release of stored T4 and T3 into the circulation, followed by transient hypothyroidism, resulting in euthyroidism or, less often, in persistent hypothyroidism [6]. Graves’ disease manifests most often as hyperthyroidism (i.e., overactive thyroid gland), but it shows an autoimmune nature, with spontaneous remissions [8,9]. Hypothyroidism without a precedent thyrotoxic phase is a distinct clinical entity or follows a thyrotoxic phase. In that respect, the continuum of ir thyroid disorders with hyperthyroidism and hypothyroidism at the two extremes is reminiscent of the continuum of thyroid disorders in the general population, but it poses distinct challenges that impose awareness.

The severity of ir thyroid disorders is classified into five grades according to the Common Terminology Criteria for Adverse Events (CTCAE): grade 1 (mild), grade 2 (moderate), grade 3 (severe or medically significant but not immediately life-threatening), grade 4 (life-threatening), and grade 5 (death) [10]. 

The present review provides a comprehensive overview of the advancements in the understanding of ir thyroid disorders. It presents the latest data concerning the biological background, the epidemiological profile, the clinical spectrum, the diagnosis, the treatment, the predictive value of antithyroid antibodies for the development of ir thyroid disorders, and the predictive value of ir thyroid disorders for the therapeutic efficacy of ICPi and/or for the improvement of the overall response and/or the overall survival (OS) and/or the progression-free survival (PFS) compared to the absence of irAEs. It builds on the existing literature, delving into the pending questions concerning ir thyroid disorders, indicating research priorities.

## 2. Why Are Ir Thyroid Disorders Important for Patients with Hematological Malignancies?

Over the last four decades, breakthrough treatments have revolutionized the survival rates of hematological malignancies, urging clinicians to prioritize the counteraction of attendant toxicities. In this context, the prevention and prompt management of ir thyroid disorders in patients with hematological malignancies is of paramount importance for many reasons [10]. 

**Figure 1:**
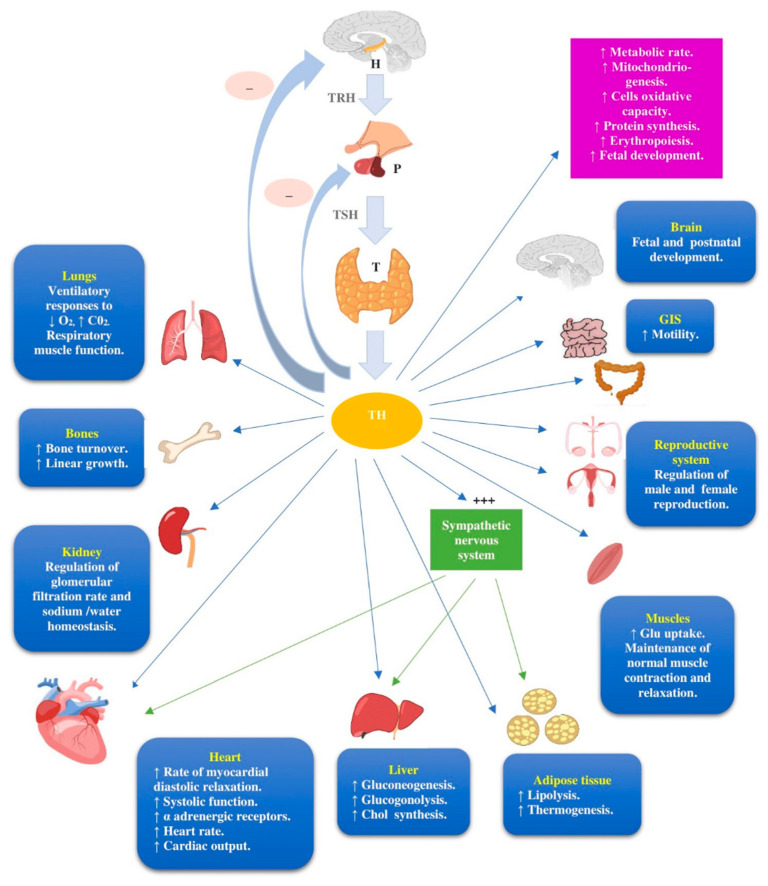
The hypothalamus–pituitary–thyroid (H–P–T) axis and the main actions of thyroid hormones (TH). The TH levels are regulated by the hypothalamic–pituitary–thyroid (HPT) axis. Thyrotropin-releasing hormone (TRH) is synthesized and released by hypothalamus (H) and stimulates the pituitary (P) gland to synthesize and secrete a thyroid-stimulating hormone (TSH), which, in turn, stimulates the thyroid (T) to synthesize and release TH. TH inhibit TRH and TSH synthesis and release through negative feedback mechanisms. TH actions affect multiple organs and tissues and physiological functions. The TH effects on the heart, liver, and adipose tissue are both direct and indirect through increased function of the sympathetic nervous system. Abbreviations: chol, cholesterol; GIS, gastrointestinal system; Glu, glucose; H, hypothalamus; P, pituitary; T, thyroid; TH; thyroid hormones; TRH, thyrotropin releasing hormone; TSH, thyroid-stimulating hormone.

First, hematologists and healthcare providers should be aware of the complex nature of the hypothalamus–pituitary–thyroid (H–P–T) axis and the pivotal role of thyroid hormones in the function of all organs and systems [11], as illustrated in Figure 1.

In the case of clinical suspicion of ir thyroid disorders, ICPi-treated patients with hematological malignancies should be promptly referred to endocrinologists. The underdiagnosis of ir thyroid disorders due to overlapping symptoms with hematological malignancies per se or with other toxicities of anticancer treatments may not only subvert the patient’s quality of life, but it can also impose dose reductions or the cessation of potentially life-saving anticancer treatments. Furthermore, untreated ir hypothyroidism can impair the pharmacokinetics and clearance of anticancer treatments other than ICPi, increasing their toxicity [12]. 

Second, the clinical significance of ir thyroid disorders, especially in patients with hematological malignancies, is highlighted by the fact that the complications of ir thyroid disorders may have an additive effect on various anticancer treatment-related organ toxicities, such as cardiac and gonadal toxicity. Additionally, ir hypothyroidism can mask other anticancer treatment-related endocrine disorders, such as latent secondary deficiency and latent central diabetes insipidus. Thus, the correction of ir hypothyroidism can trigger, respectively, an adrenal crisis and hypernatremia, with devastating consequences.

Third, the accumulating nonclinical and clinical data show that aberrant thyroid functions may influence the progression of hematological malignancies, but the precise clinical outcomes remain elusive. Indeed, both hypothyroidism and hyperthyroidism have been shown to stimulate the progression of hematological malignancies [13]. The interpretation of the discordant evidence is daunting, likely reflecting the dual effects of thyroid hormones in cancer, which can be either tumor-promoting or tumor-inhibiting [14]. Until more studies clarify this issue, the correction of ir thyroid disorders in patients with hematological malignancies and relevant survivors should be cautious and guided by clinical symptoms of hypothyroidism or hyperthyroidism in harmony with the principal “first, do no harm”.

## 3. The Biological Background of Ir Thyroid Disorders

Consolidating the complexity of immune system, the biological background of ir thyroid disorders remains elusive, mirroring the gaps in the understanding of the biological background of irAEs. The prevailing hypothesis to interpret the pathogenesis of irAEs involves an interplay among cellular autoimmunity, humoral immunity, and genetics. This hypothesis is strengthened by evidence showing: (a) a cross-reactivity of T cells (T cells against both tumor antigens and antigens in tissues that are affected by irAEs; (b) increased levels of interferon gamma (IFNγ)-inducible chemokines, such as CXCL9 and CXCL10, which are chemotactic attractors for T cells; (c) the contribution of antibody-dependent cell-mediated cytotoxicity (ADCC); and (d) the association of ir endocrinopathies with the HLA-DR allele, known to increase the risk of autoimmune endocrinopathies [15]. 

Self-reactive T-lymphocytes due to the loss of T-cell tolerance are considered key players of most irAEs, acting either directly or indirectly via stimulating the production of autoantibodies by B-lymphocytes [16]. 

Distinct mechanisms specifically underlying ipilimumab-induced autoimmunity have been suggested, namely: (a) epitope spreading, resulting in the recruitment of more T cells and stimulation of the immune response to epitopes different from the primary epitope recognized by the original effector T cells, and (b) the functional flexibility and plasticity of T cells [17]. 

The analysis of antibodies from the baseline sera of cancer patients with ICPi immunotoxicity revealed an autoimmune phenotype characterized by a toxicity-associated autoantibody profile, which was predisposed toward the development of irAEs. Protein antigens were highly expressed in tissues involved in irAEs and were implicated in pathways that correlate with immunity–autoimmunity, such as “apoptosis”, “tumor necrosis factor-a (TNF-a) signaling”, the “interleukin-1 (IL-1) pathway”, and “Toll-like receptor (TLR) signaling” [18]. 

It remains uncertain whether the correlation of polymorphisms in CTLA-4 and, less often, in PDL-1 genes with susceptibility to thyroid autoimmunity translates into the association of a CTLA-4 and PDL-1 blockade with ir thyroid disorders [19].

More insights into the biological background of ir thyroid disorders are provided by hallmark studies. An analysis of peripheral blood lymphocytes in a patient with normal thyroid function but positive antithyroid peroxidase antibodies (anti-TPOAbs) and antithyroglobulin antibodies (anti-TgAbs) at the baseline, who developed nivolumab-related hypothyroidism with increased titers of thyroid autoantibodies, demonstrated a profound elevation in the proportion of follicular helper T (Tfh) cells from 0.9% at the baseline to 3.1% 2 weeks after the initiation of nivolumab, followed by a decrease to 1.2% 4 months after the initiation of nivolumab [19]. Given that the PD-1/PD-L1 interaction transduces inhibitory signals in Tfh cells [20], it is hypothesized that anti-PD-1 mAbs reinforce the proliferation of Tfh cells, which, in turn, promote the development of anti-TPOAbs, anti-TgAbs, and thyrotropin receptor antibodies (TRAbs) [19].

Recently, Kotwal et al. characterized intrathyroidal and circulating immune cells and class II HLA in the setting of ir thyroiditis. The authors compared thyroid fine needle aspirates from nine patients and peripheral blood from seven patients with ir thyroiditis to samples of healthy thyroids from five patients and blood samples of 44 healthy volunteers, implementing flow cytometry. HLA class II was investigated in nine patients with ir thyroiditis [21]. An increase of the overall T-lymphocytes, CD4^−^CD8^−^ T-lymphocytes, and CD8^+^T-lymphocytes was observed in the ir thyroiditis samples compared to the healthy thyroid samples (61.3% vs. 20.1%, *p* = 0.00006; 1.9% vs. 0.7%, *p* = 0.006; 38.6% vs. 25.7%; *p* = 0.0259, respectively). Additionally, the anti-PD-1 mAbs-induced thyroiditis presented: (i) increased intrathyroidal CD4^+^PD-1^+^ T-lymphocytes (40.4% vs. 0.8%; *p* = 0.021) and CD8^+^PD-1^+^ T-lymphocytes (28.8% vs. 1.5%; *p* = 0.038) compared to the blood and (ii) increased circulating NK cells, CD4^+^CD8^+^ T-lymphocytes, CD4^−^CD8^−^ γΔ (gamma-delta) T-lymphocytes, and intermediate monocytes. Finally, HLA haplotypes HLA-DR4-DR53 and HLA-DR15 were identified in six and three patients, respectively. Taken together, the development of ir thyroiditis in genetically predisposed patients may be T cell-mediated [21]. 

A distinct immune phenotype of pembrolizumab-induced ir thyroid disorders was revealed by the prospective immune phenotyping of the blood of seven patients with pembrolizumab–induced thyroiditis compared to healthy volunteers and patients with autoimmune thyroid disease. Substantial elevations in CD3^+^ T-cell counts (cells/µL), both CD4^+^ and CD8^+^ T-cell subpopulations, other T-cell subsets such as *γ*Δ T cells, and CD56^+^CD3^+^ natural killer (NK) cells were observed in autoimmune patients. The T-cell counts were not elevated in pembrolizumab-treated patients. CD56^+^CD16^+^ NK cells were elevated in autoimmune and pembrolizumab-induced thyroiditis patients, while immature CD56^br^CD16^−^ NK cells were decreased in pembrolizumab-induced thyroiditis patients and elevated in autoimmune patients.

Patients with pembrolizumab-induced thyroiditis exhibited a substantial decrease in the number of immunosuppressive monocytes characterized by low HLA-DR expression (CD14^+^HLA-DR^lo/neg^ monocytes). The HLA-DR surface expression was upregulated in the inflammatory intermediate CD14^+^CD16^+^ monocytes, while no alteration in the overall number of CD14^+^CD16^+^ monocytes was observed in the peripheral blood. No major differences in the number of circulating regulatory T cells, B cells, total monocytes, granulocytes, or dendritic cells were observed among patients with pembrolizumab-induced thyroiditis, healthy volunteers, and patients with autoimmune thyroid disease. 

The absence of the expression of PD-1 on T cells from pembrolizumab-induced thyroiditis patients as opposed to PD-1 expression on T cells from autoimmune patients and healthy volunteers strengthens the hypothesis of the T cell-mediated mechanism of pembrolizumab-induced thyroiditis. On the contrary, the autoimmune setting is known to be antibody-mediated [22]. 

The identification of the cytopathologic features of ir thyroiditis may illuminate the underlying pathophysiology. Such features comprise a few lymphohistiocytic aggregates typical of Hashimoto thyroiditis and abundant clusters of necrotic cells with rare-to-absent thyroid follicular cells. Immunostaining indicated the presence of a few AE1/AE3 keratin-positive thyroid follicular epithelial cells clustered with lymphocytes and histiocytes positive for CD163—a hemoglobin scavenger receptor characteristic of macrophages of the M2 phenotype. Although CD163 is expressed both in the tumor microenvironment and in autoimmune thyroid disease, necrotic cells are not present in autoimmune thyroid disease, pointing to the distinct background of ir thyroiditis [23]. 

Recently, Neppl et al. described the histology of a thyroidectomy specimen from a patient with nivolumab-induced thyrotoxicosis and TRAb negativity, which revealed similarities with autoimmune thyroiditis, i.e., a predominance of CD8+ T cells within inflammatory infiltrates, but, also, differences from autoimmune thyroiditis, i.e., chronic thyroid lymphocytic inflammation with granulomas and the destruction of follicles [24].

**Figure 2:**
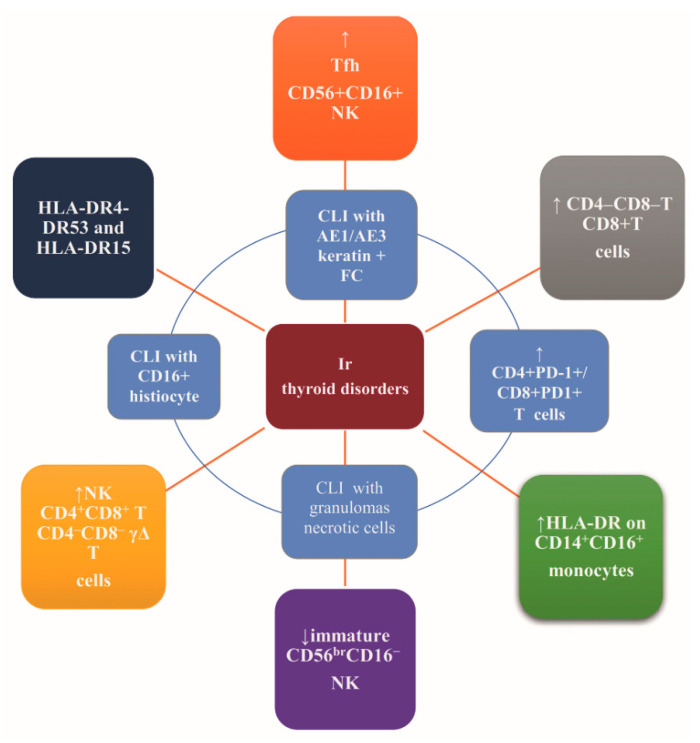
Suggested mechanisms of the biological background of ir thyroid disorders. Abbreviations: CD, cluster of differentiation; CLI, chronic lymphocytic infiltration; FC, follicular cells; *HLA*, *human leukocyte antigen;* NK, natural killers; PD-1, programmed cell death protein 1; Tfh, follicular T-helper cells.

Overall, ir thyroid disorders may be at least partially ascribed to ICPi-induced autoimmunity while constituting a distinct entity [25]. The postulation that the immunotoxicity-associated autoimmune phenotype recapitulates a crosstalk among the genetic, environmental, and tumor-specific factors remains to be explored. Additionally, a causative relationship of autoantibodies with immunotoxicity has yet to be established [25]. Figure 2 recapitulates the suggested mechanisms of the biological background of ir thyroid disorders.

## 4. The Epidemiological Profile of Ir Thyroid Disorders

The epidemiological profile of ir thyroid disorders is a rapidly evolving field of research. A plethora of reviews and meta-analyses have revealed a considerably varying incidence of ir thyroid disorders [26,27,28,29,30,31,32,33,34,35,36,37,38,39,40,41,42,43,44,45]. Of note, the terms “thyrotoxicosis” (status of excess thyroid hormones of any cause) and “hyperthyroidism” (excess thyroid hormones due to an overactive thyroid gland, e.g., Graves’ disease) are used interchangeably in many oncological studies, which may lead to the underestimation of ir Graves’ disease, because clinicians omit the conduction of laboratory tests and imaging procedures that are essential to specifying the cause of the status of excess thyroid hormones. Sensitive and specific tools for the rapid and accurate diagnosis and differential diagnosis of Graves’ hyperthyroidism are the measurement of the serum TSH-R-Ab, the color flow, or the power Doppler examination of thyroid glands that quantifies the thyroid vascularity and the thyroid scintigraphy. If these examinations are omitted, some cases of ir Graves’ disease without the typical clinical triad of ophthalmopathy, goiter, and family history may evade diagnosis. 

A recent review of 30 phase III randomized clinical trials assessing the incidence of ir thyroid disorders and 50 observational, cohort, and case studies describing the clinical features of ir thyroid disorders demonstrated the incidence of ir thyroid disorders as 4.7% for ipilimumab, 22.2% for atezolizumab, 8.8% for nivolumab, and 15.6% for pembrolizumab. Among 1436 patients treated with a combination of ipilimumab plus nivolumab, 230 patients (16.0%) developed an ir thyroid disorder. A sequential treatment of ipilimumab, then nivolumab was related to an incidence of ir thyroid disorders of 15.1%, higher than that of the ipilimumab monotherapy but similar to that of the combination treatment [37]. 

According to the meta-analysis of de Filette et al., anti-PD-1/anti-PD-L1 mAbs were related to a higher incidence of hypothyroidism compared to ipilimumab. Especially, the incidence of hypothyroidism was 8.0% (95% confidence interval (CI), 6.4–9.8) for nivolumab, 8.5% (95% CI, 7.5–9.7) for pembrolizumab, 6.0% (95% CI, 4.2–8.4) for atezolizumab, 5.5% (95% CI, 3.5–8.7) for avelumab, and 4.7% (95% CI, 2.5–8.8) for durvalumab but 3.8% for ipilimumab (95% CI, 2.6–5.5). Data on tremelimumab were missing. Combination therapies further increased the incidence of hypothyroidism, resulting in an incidence of 10.2% (95% CI, 5.6–17.9) for the combination of durvalumab with tremelimumab, 15.1% (95% CI, 10.6–21.8) for the combination of ipilimumab with pembrolizumab, and 16.4% (95% CI, 11.7–22.5) for the combination of ipilimumab with nivolumab. All ICPi significantly increased the risk of hypothyroidism compared to the placebo/chemotherapy. The pooled incidence for hyperthyroidism was 2.8% (95% CI, 2.1–3.8) for nivolumab, 3.7% (95% CI, 2.8–4.7) for pembrolizumab, 2.3% (95% CI, 0.6–8.6) for avelumab, and 1.4% (95% CI, 0.8–2.4) for ipilimumab. There was a lack of data for atezolizumab and durvalumab. Combination regimens further increased the incidence of hyperthyroidism, leading to an incidence of 9.4% (95% CI, 7.1–12.3) for ipilimumab plus nivolumab and 10.4% (95% CI, 6.6–16.1) for ipilimumab plus pembrolizumab [30].

In the meta-analysis of Barosso Sousa et al., the overall incidence of hypothyroidism was 6.6% (95% CI, 5.5–7.8%), with statistically significant differences among the classes of the ICPi regimens (*p* < 0.001). Hypothyroidism showed a predicted incidence ranging from 3.8% (95% CI, 1.9–7.8%) for ipilimumab to 13.2% (95% CI, 6.9–23.8%) for combination therapy. Both anti-PD-1 mAbs (odds ratio (OR), 1.89; 95% CI, 1.17–3.05; adjusted *p* = 0.03) and combination regimens (OR, 3.81; 95% CI, 2.10–6.91; unadjusted *p* < 0.001) were significantly more likely to elicit any grade of hypothyroidism than ipilimumab monotherapy. The difference in the risk of hypothyroidism between anti-PD-1 and anti-PD-L1 mAbs was not statistically significant (OR, 0.53; 95% CI, 0.29–0.96; adjusted *p* = 0 .11). Likewise, the difference in the risk of hypothyroidism between anti-PD-L1 mAbs and ipilimumab was not statistically significant [31]. 

In the same meta-analysis, the overall incidence of hyperthyroidism was 2.9% (95% CI, 2.4–3.7%), with statistically significant differences among the distinct classes of ICPi (*p* < 0 .001). Hyperthyroidism showed a predicted incidence ranging from 0.6% (95% CI, 0.2–1.8%) for anti-PD-L1 mAbs to 8.0% (95% CI, 4.1–15.3) for combination regimens. Compared to ipilimumab, combination regimens were significantly more likely to elicit any grade of hyperthyroidism (OR, 4.27; 95% CI, 2.05–8.90; *p* = 0.001), whereas anti-PD-1 mAbs did not increase the incidence of hyperthyroidism statistically significantly after correcting for multiple comparisons (OR, 1.89; 95% CI, 1.02–3.52; adjusted *p* = 0.13). The risk of anti-PD-1 mAbs-induced hyperthyroidism was significantly higher compared to that of anti-PD-L1 mAbs-induced hyperthyroidism (OR, 5.36; 95% CI, 2.04–14.08; adjusted *p* = 0.002) [31]. None of the 38 studies included in this systematic review and meta-analysis determined the etiology of thyrotoxicosis [31].

A recent systematic review and meta-analysis addressing the irAEs of anti-PD-1 and anti-PD-L1 mAbs, including relevant information from ClinicalTrials.gov, showed that hypothyroidism was the most common organ-specific adverse event among patients exposed to anti-PD-1 mAbs. Increased rates of hypothyroidism were observed with anti-PD-1 mAbs compared to the standard treatment (OR, 7.56, 95% CI, 4.53–12.61). Patients treated with anti-PD-1 mAbs were at a higher risk for any grade of hypothyroidism (OR, 6.92, 95% CI, 3.25–14.75, *p* < 0.001) [28].

Another systematic review and meta-analysis showed that the hyperthyroidism of any grade ranks second among endocrine irAEs, following hypophysitis, affecting 100 patients (2.5%), while the hypothyroidism of any grade ranks third, affecting nine patients (0.2%). Patients treated with anti-CTLA-4 mAbs (ipilimumab and tremelimumab) had higher risks of hypothyroidism (OR, 7.86, 95% CI, 4.10–15.04) but not hyperthyroidism (OR, 3.78, 95% CI, 0.94–15.17), compared to the control groups receiving a placebo, chemotherapy, radiation therapy, or vaccine. The same trends were observed in terms of severe ir thyroid disorders [29].

A meta-analysis comparing the safety of ICPi monotherapy to that of ICPi combination regimens, including 10 studies involving eight randomized controlled trials with 2716 patients, demonstrated that the ICPi combination regimens significantly increased the risk of any grade of hypothyroidism (relative risk (RR), 2.17; 95% CI, 1.71–2.76; *p* < 0.05), and hyperthyroidism (RR, 3.13; 95% CI, 2.08–4.70; *p* < 0.05) [35].

Table 1 and Table 2 recapitulate the frequency of ir thyroid disorders according to the most recent systematic reviews and meta-analyses [26,27,28,29,30,31,32,33,34,35] (Table 1) and the most recent reviews [36,37,38,39,40,41,42,43,44,45] (Table 2). 

Taken together, across the studies depicted in these two tables, the incidence of hypothyroidism ranges from 0% [45] to 11% [41]) for anti-CTLA-4 mAbs, from 2.5% [44] to 10.19% [26] for anti-PD-1/anti-PD-L1 mAbs, and from 4% to 27% [44] for combination regimens. The incidence of thyrotoxicosis ranges from 0% [26,29] to 4% [44] for anti-CTLA-4 mAbs, from 0.4% to 7.8% for anti-PD-1/anti-PD-L1 mAbs [44], and from 4.3% to 14% for combination regimens [44].

The path forward is to move beyond the systematic reanalysis of the existent literature to embrace evidence-based medicine guided by real-world data. Such real-world data from seminal pharmacovigilance studies are depicted in Table 3 [5,7,46,47,48]. 

According to a real-world study leveraging FAERS from the first quarter of 2014 to the first quarter of 2019 by Zhai et al., hypothyroidism ranks first and hyperthyroidism ranks fourth among the endocrine irAEs, affecting, respectively, 885 patients (14.14%) and 472 patients (7.54%) [46]. This study showed a much stronger association of hypothyroidism with anti-PD-1 mAbs monotherapies, especially nivolumab or pembrolizumab, and with the combination of nivolumab pus ipilimumab. Hyperthyroidism was more strongly associated with anti-PD-1 mAbs monotherapies, especially nivolumab or durvalumab, and with the combination of nivolumab and ipilimumab [46].

According to the largest and most extensive to date, the analysis of ir thyroid disorders based on the WHO Global Database by Bai et al., anti-PD-1/anti-PDL-1 mAbs demonstrate a higher risk of developing thyroid disorders than anti-CTLA-4 mAbs. The risk is higher with a ICPi combination than a monotherapy, especially for a thyrotoxic crisis (reporting odds ratio (ROR), 11.45; 95% CI 2.44–53.84). Most of the reports of hypothyroidism (578 cases, 51.38%), hyperthyroidism (447 cases, 48.27%), and thyroiditis (128 cases, 43.54%) [47] were associated with nivolumab. 

A search of the French Pharmacovigilance database for irAEs induced by nivolumab, pembrolizumab, and ipilimumab reported before April 2017 revealed an incidence of peripheral thyroiditis (thyrotoxicosis and hypothyroidism) of 44.2%, with hypothyroidism and thyrotoxicosis being equally reported. The cases related to nivolumab and pembrolizumab were the most common, representing, respectively, 52.7% and 35.5%. Nivolumab and pembrolizumab seemed to be preferentially associated with hypothyroidism compared to ipilimumab [48]. 

It is acknowledged that pharmacovigilance studies have not proven a causal relationship between ICPi and ir thyroid disorders; however, they have provided fuel for clinical research hypotheses.

Interestingly, certain clinical studies have revealed rates of ir thyroid disorders higher compared to systematic reviews and meta-analyses. For instance, in the study of Morganstein et al., enrolling 190 consecutive melanoma patients treated with ICPi, 56 patients (30%) developed thyroid disorders, with rates 23%, 39%, and 50% among the patients on, respectively, an ipilimumab monotherapy, anti-PD-1 monotherapy, and combination of ipilimumab with nivolumab [49]. The study of 1146 individuals without pre-existing thyroid disease who received ICPi between 2012 and 2018 from the Electronic Health Record of a large U.S. academic center showed an overall incidence of ir thyroid disease of 19%, specifically 13.4% for hypothyroidism and 9.5% for hyperthyroidism. Surprisingly, no significant association between ir thyroid disorders and a specific ICPi was observed in the overall analysis. Ir thyroid disorders were more common in patients who received a combination of ipilimumab plus nivolumab (31%) compared to patients who received a pembrolizumab (18%, *p* = 0.03), nivolumab (18%, *p* < 0.01), or ipilimumab (15%, *p* = 0.02) monotherapy [50]. In a cohort of 470 patients who received ICPi for metastatic renal and urothelial cancer, hypothyroidism was the most common irAE, with an incidence of 22.6% [51]. 

There is a paucity of data about thyroid disorders related to cemiplimab, a novel anti-PD-1 mAb that received FDA approval in September 2018 for the treatment of metastatic or locally advanced cutaneous squamous cell carcinoma not amenable to surgery or radiation [52]. According to the summary of the information on cemiplimab, ir hypothyroidism and ir hyperthyroidism of any grade are common, with incidences of 9.6% and 2.7%, respectively, while ir thyroiditis is uncommon, with an incidence of 0.2% (any grade) [53]. No ir thyroid disorder of grades 3–5 was recorded. The primary analysis of a pivotal Phase II study of a cohort of patients with metastatic cutaneous squamous cell carcinoma (NCT02760498) treated with cemiplimab demonstrated an incidence of hypothyroidism of any grade of 8%, but no hypothyroidism grade ≥3 was observed [54]. 

Ir thyroid disorders appear to be more frequent among older patients. In the cohort of ir thyroid disorders identified in the scoping review of Tan et al., the median age of the patients was 54.5 for thyrotoxicosis due to Graves’ disease, 61 years for thyrotoxicosis due to thyroiditis, 64 years for primary hypothyroidism, and 63 years for hypothyroidism with precedent thyrotoxicosis [38].

In the analysis of the French Pharmacovigilance database addressing the irAEs of nivolumab, pembrolizumab, and ipilimumab reported before 30 April 2017, the mean age of the patients with ir thyroid disorders was 60.2 years, with the youngest patient being 22 years old and the oldest patient being 87 years old [48]. On the other hand, a review of 30 phase III randomized clinical trials and 50 observational, cohort, and case studies revealed no age predominance in terms of ir thyroid disorders [37]. The same review also revealed no sex disparity in terms of ir thyroid disorders [37]. Likewise, the male-to-female ratio of ir thyroid disorders was approximately one in the analysis of the French Pharmacovigilance database addressing the irAEs of nivolumab, pembrolizumab, and ipilimumab reported before 30 April 2017 [48]. However, in a comprehensive retrospective review from a single institution conducted by Ryder et al., the male-to-female ratio of hypothyroidism was 6:9. Especially, the male-to-female ratio of ipilimumab was 2:4 concerning hypothyroidism related to the combination of ipilimumab with nivolumab [39]. According to the pharmacovigilance study of Zhai et al., males present with significantly lower reporting frequencies of hypothyroidism (ROR = 0.68, 95%CI, 0.59–0.78) and hyperthyroidism (ROR = 0.77, 95%CI, 0.63–0.93) [46]. However, in the scoping review of Tan et al., there was a male predominance regarding ir Graves’ disease and ir thyrotoxicosis evolving into hypothyroidism but no sex differences regarding thyroiditis and primary hypothyroidism [38]. The largest and most extensive analysis of the clinical characteristics of ir thyroid disorders to date based on the WHO Global Database showed a male predominance. Whether the male predominance of ir thyroid disorders reflects the predominance of male cancer patients is to be clarified [47].

In a recent study of 1146 individuals who received ICPi between 2012 and 2018 from the Electronic Health Record of a large U.S. academic center, the cancer type was significantly associated with the ir thyroid disorders after adjustment for sex and age (*p* = 0.01), with the rates of the ir thyroid disorders ranging from 10% in glioblastoma to 40% in renal cell cancer [50]. The review of Muir et al. indicated melanoma as the cancer type most often associated with ir thyroid disorders, followed by lung cancer and renal cell cancer [37]. In the scoping review of Tan et al., melanoma was responsible for most cases of primary hypothyroidism or thyrotoxicosis evolving into hypothyroidism or thyroiditis and with all cases of Graves’ disease [38].

To sum up, the variability of the current epidemiological data concerning ir thyroid disorders is remarkable and difficult to interpret. It can be speculated that this variability reflects the methodological issues and/or fundamental differences in terms of the toxicities among distinct ICPi classes and subclasses.

## 5. The Natural History of Ir Thyroid Disorders

The natural history of ir thyroid disorders is unpredictable. The onset of ir thyroid disorders varies considerably from 7 days to 3 years after ICPi initiation [39,55]. Of note, an exceptional case of Graves’ disease 8 years after treatment with tremelimumab was reported in a 55-year-old man with metastatic melanoma [56].

Hypothyroidism without a precedent thyrotoxic phase was reported 6–8 weeks after the initial exposure to ICPi [57]. In the scoping review of Tan et al., including 152 cases of ir thyroid disorders, the median time of onset was 7 weeks for thyrotoxicosis due to Graves’ disease, 6 weeks for thyrotoxicosis due to thyroiditis, 12 weeks for primary hypothyroidism, and 6 weeks for hypothyroidism with precedent thyrotoxicosis [38]. In a review of 30 phase III randomized clinical trials and 50 observational, cohort studies, and case studies by Muir et al., thyrotoxicosis occurred most often within 3–6 weeks after ICPi initiation. The earliest recorded time of onset of thyrotoxicosis was 9 days after ICPi initiation, although subclinical hyperthyroidism was diagnosed as early as 24 h after ICPi initiation. On the other hand, the onset of thyrotoxicosis later than 1 year after ICPi initiation was reported. The combination of ipilimumab plus nivolumab was demonstrated to elicit thyrotoxicosis earlier compared to anti-PD-1 mAbs [37].

In the review of 30 phase III randomized clinical trials and 50 observational, cohort studies, and case studies by Muir et al., the thyrotoxic phase was usually asymptomatic and self-limited, requiring no therapeutic intervention, with an average duration of 4–6 weeks [37]. Alarmingly, destructive thyrotoxicosis can evolve into hypothyroidism rapidly. For instance, thyroid-stimulating hormone (TSH) levels >50 mIU/mL were found as early as 18–28 days after the last normal TSH value in two patients with transient hyperthyroidism progressing rapidly into hypothyroidism [58].

In a retrospective cohort analysis of patients treated with either a combination therapy or monotherapy, the initial presentation of ir thyroid disorders was hypothyroidism in only 22% of patients. While thyrotoxicosis was frequently followed by hypothyroidism, no case of hypothyroidism was followed by thyrotoxicosis. Eighty percent of the patients with thyrotoxicosis subsequently developed hypothyroidism. The percentage of patients with thyrotoxicosis as the initial presentation was significantly higher in the combination therapy group than in the monotherapy group (93% vs. 56%; *p* = 0.01). Eighty-three percent of the cases of thyrotoxicosis related to a combination therapy and 36% of the cases of thyrotoxicosis related to a monotherapy were already diagnosed within 21 days after the first infusion of the regimens. Within 84 days after the first treatment, 100% of the cases of thyrotoxicosis related to a combination therapy and 90% of the cases of thyrotoxicosis related to a monotherapy were diagnosed. The median time to the onset of hypothyroidism was 63 days (range 24–141 days) and 70 days (range 27–475 days) in the combination and monotherapy groups, respectively (*p* = 0.09). A combination therapy accelerated the onset of hypothyroidism with precedent thyrotoxicosis (median time 63 days) and hypothyroidism only (median time 26 days) compared to a monotherapy (median times 84 days and 56 days, respectively). The median time to the onset of hypothyroidism as the initial presentation was shorter in the case of either a monotherapy or combination therapy, with no significant differences. The median time from the onset of thyrotoxicosis to the onset of hypothyroidism was 42 days (range 14–169), with no significant differences between the combination therapy and monotherapy [55].

In a retrospective study enrolling cancer patients with normal baseline thyroid function tests who developed ir painless thyroiditis, the median time of onset of thyrotoxicosis was 5.3 weeks (range 0.6–19.6 weeks) after the initiation of ICPi. The median duration of thyrotoxicosis was 6 weeks (range 2.6–39.7 weeks). The incidence of ir hypothyroidism was 84%, with a median of 10.4 weeks (range 3.4–48.7 weeks) after the initiation of ICPi. Ipilimumab plus nivolumab vs. nivolumab alone accelerated the development of thyrotoxicosis (median time of onset 2 weeks vs. 6 weeks after the ICPi initiation; *p* = 0.26) and hypothyroidism (median time of onset 10 weeks vs. 17 weeks after the ICPi initiation; *p* = 0.029) [59].

Recently, a descriptive, retrospective, and multicenter study of the patients with ir thyroid disorders showed that 80% of the patients experienced a switch from ir hyperthyroidism to ir hypothyroidism during their follow-up. The most common ir thyroid disorder was hypothyroidism (57%) in the monotherapy group, as opposed to hyperthyroidism (77%) in the combination group (*p* = 0.002). The onset of an ir thyroid disorder was significantly accelerated by a combination treatment compared to a monotherapy (*p* = 0.001) [60].

According to a review of 30 phase III randomized clinical trials and 50 observational, cohort, and case studies by Muir et al., overt hypothyroidism without a precedent thyrotoxic phase may be the initial ir thyroid disorder, with a variable incidence (10–60%) and a time to onset typically longer than that of thyrotoxicosis [37].

The largest and most extensive analysis of the clinical characteristics of ir thyroid disorders to date based on the WHO Global Database by Bai et al. revealed an earlier time of onset of hypothyroidism related to anti-CTLA-4 mAbs compared to anti-PD mAbs, but the time of onset of hyperthyroidism was similar for these two treatments. The median duration of thyrotoxicosis was 36 days (interquartile range (IQR), 9–63). The authors postulated that the short duration of thyrotoxicosis may lead to an underestimation of the number of relevant cases [47].

Taken together, thyroid disorders are generally considered as a continuum, with hyperthyroidism and hypothyroidism at two extremes for two reasons. First, thyrotoxicosis can be the initial manifestation of destructive thyroiditis, which may result in hypothyroidism either transient or permanent. Second, hyperthyroidism due to Graves’ disease can eventually result in hypothyroidism. Most importantly, we emphasize this continuum especially in the setting of ir thyroid disorders, because ir thyrotoxicosis as the initial phase of ir destructive thyroiditis has been reported to remain asymptomatic in most cases (67%), evading a diagnosis [59]. Accordingly, ir hypothyroidism without a clinically evident precedent thyrotoxic phase may be part of the natural course of destructive thyroiditis or it may be ascribed to the de novo development of autoimmune hypothyroidism. Thus, we suggest that, considering the continuum of ir thyroid disorders, clinicians need to closely monitor the thyroid function in ICPi-treated patients and personalize their diagnostic procedures and therapeutic interventions.

## 6. The Clinical Presentation of Ir Thyroid Disorders

The clinical presentation of ir thyroid disorders is multifaceted. The main clinical manifestations of hypothyroidism are impaired mental activity, weight gain, fatigue, cold intolerance, depression, weakness, dry skin, alopecia, puffiness, constipation, bradycardia, and a delayed relaxation of tendon reflexes [61]. The diagnosis of thyrotoxicosis is often indicated by unequivocal clinical manifestations: namely, anxiety, emotional lability, weakness, tremor, palpitations, heat intolerance, increased perspiration, and weight loss despite a normal or increased appetite. Additional symptoms and signs that raise suspicion of thyrotoxicosis are new onset atrial fibrillation, myopathy, hyperdefecation (not diarrhea), urinary frequency, oligomenorrhea or amenorrhea in women, and gynecomastia and erectile dysfunction in men [62].

In the study of Lee et al., the most common symptoms of ir hypothyroidism were fatigue and weight gain. Among patients with recorded weights, a weight gain was observed in 89% of patients receiving a combination therapy and in 80% of patients receiving a monotherapy. The most common symptoms of patients with ir thyrotoxicosis were weight loss and tachycardia. Neck pain was reported in two patients. Among patients with recorded weights, a weight loss was observed in 90% of patients receiving a combination therapy and in 89% of patients receiving a monotherapy. Among patients with a recorded heart rate, tachycardia (heart rate > 90 bpm) was observed in 38% of patients receiving a combination therapy and in 29% of patients receiving a monotherapy [55].

Alarmingly, the prevailing clinical manifestations of either hypothyroidism (fatigue, weight gain, or bradycardia) or thyrotoxicosis (fatigue, nervousness, weight loss, or palpitations) may be misinterpreted as symptoms of the underlying malignancy or overshadowed by symptoms of the underlying malignancy [55].

An analysis of the French Pharmacovigilance database for ir thyroid disorders induced by nivolumab, pembrolizumab, and ipilimumab reported before April 2017 revealed that almost half of the patients (47.2%) were asymptomatic [48]. Likewise, in a retrospective study of 657 cancer patients treated with ICPi between November 2014 and July 2016 at the MD Anderson Cancer Center and referred to an endocrinologist due to suspicion of ir painless thyroiditis, 67% of the patients were asymptomatic during the thyrotoxic phase [59]. The reason for the absence of early symptoms after a remarkable elevation of their TSH levels in ir hypothyroidism or of their fT4 levels in ir thyrotoxicosis could be the fact that acute changes in thyroid hormones may take time to translate into alterations of the gene expression (genomic functions of thyroid hormones) [62].

### Remarkable Clinical Aspects of ir Thyroid Disorders

A distinct inflammatory condition called thyroid eye disease (TED)-like orbital inflammatory syndrome with either normal thyroid function or hyperthyroidism is being increasingly correlated with ICPi [63,64,65,66,67,68,69]. The presentation of ICPi-induced ophthalmopathy is reminiscent of that of Graves’ disease, comprising proptosis, eye pain, conjunctival redness, periorbital edema, ophthalmoplegia, and swelling of the extraocular muscles in an MRI. The epidemiologic data point to the association of variant polymorphisms of the CTLA-4 receptor gene with ICPi-induced thyroid eye disease [66].

An exceptional case of ir hypothyroidism-related myopathy was described in a young patient with surgically resected stage IIIB melanoma 12 weeks after the initiation of nivolumab. The myopathy manifested as severe myalgias, arthralgias, intermittent disequilibrium, and a significant elevation of creatine kinase. Concomitant increased TSH levels alongside undetectable free T4 (fT4) levels pointed to hypothyroidism-related myopathy. Thyroid hormone replacement therapy led to the improvement of both the myopathy and laboratory parameters over three weeks [70].

Severe ir thyroid disorders are rare [26,28,55]. According to a systematic review and a meta-analysis addressing irAEs of anti-PD-1 mAbs overall and compared to the control treatments, severe hypothyroidism occurred in 0.2% of patients treated with anti-PD-1 mAbs [28]. Another systematic review and meta-analysis addressing irAEs associated with an ICPi monotherapy or combinations in advanced melanoma patients revealed an incidence of grade ≥3 ir hypothyroidism of 0% for ipilimumab, nivolumab, pembrolizumab, and the combination of ipilimumab with pembrolizumab and of 0.08% for the combination of ipilimumab with nivolumab. The incidence of grade ≥3 ir hyperthyroidism was 0.10% for ipilimumab, 0% for either pembrolizumab or nivolumab, 1.31% for the combination of ipilimumab with pembrolizumab, and 0.66% for the combination of ipilimumab with nivolumab [26].

Only a few cases of life-threatening thyroid storm [71,72,73] or myxedema coma [74,75] have been reported. The first analysis of a thyrotoxic crisis based on the WHO Global Database revealed 11 cases of thyrotoxic crisis, all of which occurred in malignant melanoma patients. Most of the cases of thyrotoxic crisis were associated with ICPi combination therapy (seven cases, 63.64%). Notably, seven cases of a thyrotoxic crisis recovered. Due to the severity of a thyrotoxic crisis, the authors recommended an assessment of their thyroid function once a week, especially within 1 month after the initiation of an ICPi combination.

The median fatality rate of ir thyroid disorders is 0.15–0.93% [9] or, according to others, 0.68% [47]. Ir thyroid disorders are not a common cause of the presentation of ICPi-treated patients in the emergency department (ED) of cancer centers [76]. Nevertheless, severe hypothyroidism should be considered during the diagnostic approach to ICPi-treated cancer patients with confusion, lethargy, bradycardia, hyponatremia, or hypothermia [77]. A thyroid storm is a multisystem disorder with a high mortality rate if unrecognized and inadequately treated (10%). Clinical suspicion of a thyroid storm should be raised in ICPi-treated cancer patients presenting any evidence of systemic decompensation. A point scale for the diagnosis of a thyroid storm has been suggested based on precise criteria, including tachycardia, arrhythmias, congestive heart failure, hypotension, hyperpyrexia, agitation, delirium, psychosis, a stupor, and a coma, as well as nausea, vomiting, diarrhea, and hepatic failure [8,78].

The coexistence of ir thyroid disorders with other irAEs is being increasingly reported. Especially, thyroiditis plus pneumonitis, thyroiditis plus hepatitis, and thyroiditis plus dermatitis ranked first, second, and fourth among the multisystem irAEs patterns, with incidences of 14%, 10%, and 8%, respectively, according to a recent retrospective cohort study in five academic institutions worldwide, enrolling 623 patients with stage III/IV NSCLC treated with anti-PD-(L)1 mAbs [79].

Overall, most cases of ir thyroid disorders are mild, but the lack of specificity of symptoms imposes a heightened level of clinical suspicion to preclude life-threatening consequences of the rare but very severe cases.

## 7. Diagnostic Evaluation of Ir Thyroid Disorders

To rule out any preexisting thyroid disorder, the measurement of TSH and fT4 is recommended before the initiation of ICPi. After the initiation of ICPi, the monitoring of TSH and fT4 is recommended every 4–6 weeks according to the American Society of Clinical Oncology (ASCO) guidelines [80] or at each course of treatment for 6 months, every second course of treatment for the following 6 months, and then in the case of clinical suspicion of thyroid disorder, according to the French endocrine society guidance on endocrine side effects of immunotherapy [6].

Elevated levels of TSH with low-to-normal fT4 levels set the diagnosis of primary hypothyroidism, while low fT4 levels with inappropriately low-to-mid-normal levels of TSH set the diagnosis of secondary hypothyroidism due to pituitary dysfunction. Of note, in the case of secondary hypothyroidism, the evaluation of the remaining pituitary hormones is mandatory.

Thyrotoxicosis as initial phase of destructive thyroiditis, and hyperthyroidism in the setting of Graves’ disease is characterized by suppressed TSH levels and elevated fT4 and/or T3 levels [8,78,80]. Abnormal results of thyroid function tests are difficult to interpret in acutely ill inpatients, because they could be a mere manifestation of non-thyroidal illness syndrome (NITS) [61,81]. Additionally, exogenous glucocorticoids, a common practice in oncological patients, may suppress TSH [61].

Graves’ disease is differentially diagnosed from thyrotoxicosis ascribed to destructive thyroiditis based on positive TRAbs, a high tracer uptake on scintigraphy, and a high blood flow in a Doppler flow ultrasound (US) called “thyroid inferno” in the former, as opposed to negative TRAbs, a low uptake on scintigraphy, and the hypovascularization of thyroid parenchyma in an US in the case of painless thyroiditis [8,78]. While interpreting the results of thyroid scintigraphy, physicians should be aware of the recent administration of iodine-containing contrast media and antithyroid drugs [82,83].

Albeit not recommended as a routine diagnostic tool, diffused increased ^18^fluorodeoxyglucose uptake on positron emission tomography (^18^FDG-PET) on the thyroid gland has been reported in 64% of patients with pembrolizumab-induced thyroiditis [22] and in two patients with nivolumab-induced transient thyrotoxicosis [84]. This finding points to inflammatory thyroiditis, but it lacks specificity, being also observed in Graves’ disease and incidental chronic lymphocytic thyroiditis [85,86]. In a retrospective cohort study enrolling 200 patients treated with nivolumab, an increased ^18^FDG uptake on ^18^FDG-PET in the thyroid gland before the initiation of nivolumab was related to high incidences of overt, but not subclinical, ir thyroid disorders (adjusted odds ratio 14.48; 95% CI, 3.12–67.19) [87].

Elevated thyroglobulin (Tg) levels during the phase of thyrotoxicosis and decreasing during the subsequent hypothyroidism have been observed in nivolumab-induced destructive thyroiditis [88].

To sum up the gold standard for the diagnosis of ir thyroid disorders is the evaluation of the serum levels of TSH and of fT4, while the measurements of serum autoantibodies and the imaging procedures are individualized.

## 8. Treatment of Ir Thyroid Disorders

Several guidelines for the treatment of ir thyroid disorders, as depicted in Table 4, could serve as the initiating point for a personalized approach guided by clinical judgement.

In the case of ir thyrotoxicosis either in the setting of Graves’ disease or destructive thyroiditis, the administration of beta-blockers is recommended by the ASCO [80], European Society for Medical Oncology (ESMO) [91], SITC [89], and NCCN [90] guidelines to alleviate the symptoms of excess thyroid hormones. In the case of Grave’s disease, the relevant therapeutic protocols are applied [8,78], usually starting with thionamides, according to the NCCN and ESMO guidelines [90,91]. If ir thyrotoxicosis progresses to hypothyroidism, thyroid hormone replacement may be required, especially in cases of TSH *>* 10 mIU/l [90].

The administration of steroids is recommended by the ESMO [91] and ASCO [80] guidelines, especially prednisone 1 to 2 mg/kg/d or the equivalent tapered over 1 to 2 weeks [80] in the case of severe symptoms or a thyroid storm or inflammatory thyroiditis [91]. However, high doses of glucocorticoids (HDG), defined as a dose higher than 7.5-mg prednisone or the equivalent daily for more than 1 week, demonstrated no significant differences from no administration of HDG in terms of the median duration of thyrotoxicosis (28 vs. 42 days, respectively), median time of evolution of thyrotoxicosis to hypothyroidism (39 vs. 42 days, respectively), median time to onset of hypothyroidism (63 (range: 21–190) vs 63 (range: 14–489) days, respectively), and median maintenance dose of levothyroxine (1.5 μg/kg/day vs. 1.3 μg/kg/day, respectively) [93].

Grade 1 hypothyroidism or hyperthyroidism does not impose a withdrawal of ICPi treatment provided that the thyroid function is monitored every 2 weeks according to the ASCO and SITC guidelines [80,89] or 4–6 weeks according to the NCCN guidelines [90]. ICPi treatment may be held in grade 2 hypothyroidism [80], while it should be interrupted until the resolution of the symptoms in grades 3 and 4 hypothyroidism [80,89,90,91]. In grade 2 (ASCO guidelines) [80] or grade ≥3 (SITC guidelines) [89] hyperthyroidism, holding ICPi until the resolution of the symptoms is needed.

A prospective study addressing ir endocrinopathies in melanoma patients treated with ipilimumab or anti-PD-1 mAbs (nivolumab or pembrolizumab) or a combination of ipilimumab with anti-PD-1 mAbs showed that all patients with hypothyroidism or subclinical hypothyroidism required ongoing treatment with L-thyroxine. The median time to the resolution of hyperthyroidism was 50 ± 21 days for patients treated with antithyroid drugs and 40 ± 122 days for patients not treated with antithyroid drugs (no statistical significance; *p*>0.05) [94]. In this study, the use of antithyroid drugs did not shorten the median time to the resolution of hyperthyroidism. Albeit apparently surprising, this result is rational for two reasons. First, it reflects the intriguing natural history of Graves’ disease. Given its autoimmune nature, Graves’ disease shows a fluctuating activity with occasional spontaneous remissions even without any therapy [8,9]. In fact, Graves’ disease can be mild and self-limiting. In most cases of Graves’ disease (60–70%), the course of the disease is long, with relapses and remissions, while, in 30–40% of cases, patients experience only one episode of hyperthyroidism. Only a few patients (less than 10%) experience no remission of hyperthyroidism [9]. Second, it is highlighted that antithyroid drugs are not an etiological treatment of Graves’ disease. In fact, some novel etiological treatments for Graves’ disease are currently under evaluation [8]. In other terms, the lower mean time for the resolution of Graves’ disease in the “no antithyroid drugs group” compared to the “antithyroid drugs group” can be explained by the self-limiting nature of Graves’ disease. However, the treatment with antithyroid drugs seems to decrease the variance among the patients, lowering the standard deviation compared to the “no antithyroid drugs group”, which means that it was beneficial for certain patients. This finding highlights the need for the individualization of decision-making to preclude the overtreatment of Graves’ disease.

Overt hypothyroidism is most often irreversible, requiring long-term thyroid hormone replacement, while subclinical hypothyroidism shows a higher potential for recovery [37].

In a retrospective study enrolling cancer patients with normal baseline thyroid function tests who developed ir painless thyroiditis, L-thyroxine was required for a median follow-up of 57.4 weeks (range 1–156.7 weeks) from hypothyroidism onset. The restoration of thyroid function without L-thyroxine was observed in four patients at a median follow-up of 11.35 months (range 4.43–14.43 months) [59].

In a recent descriptive, retrospective, and multicenter study of patients with ir thyroid disorders, the continuation of L-thyroxine was required for half of the patients with ir hypothyroidism after a follow-up of 205 days (range 112–360) [60]. In the largest, to date, analysis of ir thyroid disorders based on the WHO Global Database, there were more cases of recovery from hyperthyroidism and thyroiditis than from hypothyroidism. Specifically, 59.81% of hypothyroidism, 78.35% of hyperthyroidism, and 75.16% of thyroiditis recovered, with statistically significant differences between hypothyroidism and hyperthyroidism (*p* < 0.0001) and between hypothyroidism and thyroiditis (*p* = 0.002) [47].

To promptly detect a recovery of ir hypothyroidism, the tapering of thyroid hormone replacement under an endocrinologist’s consultation and retesting of the thyroid function is recommended [80]. In the case of thyrotoxicosis, thyroid function tests can detect promptly: (i) an evolution of ir thyrotoxicosis due to destructive thyroiditis or due to Graves’ disease (rarely) [30] to ir hypothyroidism, (ii) a recovery of Graves’ disease, and (iii) a recovery of thyrotoxicosis due to destructive thyroiditis [80].

Taken together, the therapeutic interventions in the case of ir thyroid disorders in cancer patients should be individualized. The aim is to correct any clinically significant impairment of thyroid function, but clinicians need to rely on their clinical judgement to avoid either undertreatment or overtreatment.

## 9. The Contribution of Antithyroid Antibodies to Ir Thyroid Disorders

To elucidate the role of thyroid autoantibodies in ir thyroid disorders, two critical questions should be answered. Can the positivity of thyroid autoantibodies at the baseline predict the development of ir thyroid disorders? Is the positivity of thyroid antibodies at the time of diagnosis of ir thyroid disorders causatively associated with ir thyroid disorders or constitute an innocent bystander? The current relevant data are inconclusive, pointing to either the immunologic or nonimmunologic mechanistic underpinnings of ir thyroid disorders.

In a case series of 10 patients with ir hypothyroidism (related to anti-PD-1 mAbs either as a monotherapy or combined with anti-CTLA-4 mAbs), thyroiditis was initially characterized by elevated anti-TgAbs in 40% of patients and elevated TRAbs in 40% of patients. All titers decreased during the hypothyroid phase, although hypothyroidism persisted in 80% of cases [88].

In a retrospective study enrolling cancer patients with normal baseline thyroid function tests who developed ir painless thyroiditis, anti-TPOAbs and anti-TgAbs were positive in 45% and 33% of patients, respectively, at the time of diagnosis [59].

Tanaka et al. reported a case series of 14 nivolumab-treated cancer patients, three of whom developed ir thyroid disorders with varying statuses of thyroid autoantibodies. The first patient was a 70-year-old woman with unresectable vaginal melanoma who presented with normal thyroid function despite positive anti-TPOAbs and anti-TgAbs before the initiation of nivolumab. After the first dose of nivolumab, the patient developed thyrotoxicosis, which evolved into hypothyroidism with a persistence of anti-TPOAbs and anti-TgAbs positivity after the third dose of nivolumab. The second patient was a 64-year-old man with stage IV melanoma presenting subclinical hypothyroidism with negative thyroid autoantibodies before the administration of nivolumab, which evolved into clinical hypothyroidism with anti-TPOAbs positivity after the fifth injection of nivolumab. The third patient was an 80-year-old man with unresectable mucosal melanoma of the upper lip and palate presenting normal thyroid function and negative thyroid autoantibodies before the initiation of nivolumab. Following two injections of nivolumab, the patient developed thyrotoxicosis, but the negativity of thyroid autoantibodies persisted [95].

The positivity of anti-TPOAbs and anti-TgAbs proved to be a significant predictor of primary hypothyroidism in a retrospective study enrolling 64 patients with non-small-cell lung cancer (NSCLC) treated with nivolumab [96].

Ninety-nine patients with advanced melanoma (ages 26.3–93.6 years; 63.6% females) who received at least one dose of pembrolizumab were monitored prospectively within an expanded access program at a referral oncology center. Seventeen patients developed 18 adverse events of thyroid impairment. Twelve patients developed thyrotoxicosis, of whom nine patients eventually presented hypothyroidism. Isolated hypothyroidism occurred in six patients. Ten out of fifteen hypothyroid patients received thyroid hormone replacement. Thyroid autoantibodies (anti-TPOAbs and/or TRAbs) during ir thyroid disorders were assessed in 10 out of 17 patients and were found elevated in four out of 10 patients (40%). The anti-TPOAbs levels were elevated in three patients, while the TRAbs levels were elevated in one patient. In the latter patient, thyrotoxicosis evolved into hypothyroidism, pointing to a shift of Graves’ disease into hypothyroidism, likely due to a switch in the antibody subpopulation. The baseline anti-TPOAbs status was evaluated in four patients, of whom two patients had positive anti-TPOAbs at the baseline and during thyroid impairment, whereas two patients had negative anti-TPOAbs at the baseline and during thyroid impairment [30].

In a case series of 10 patients with painless thyroiditis related to anti-PD-1 mAbs, six patients developed transient thyrotoxicosis with a negativity of thyrotropin-binding inhibitory immunoglobulins (TBII), as opposed to a positivity of antithyroid antibodies in four of them. Thyrotoxicosis was managed with the administration of beta-blockers and evolved into hypothyroidism. Four patients developed hypothyroidism with the absence of a precedent thyrotoxic phase 6–8 weeks after anti-PD-1 mAbs initiation, characterized by positive antithyroid antibodies and a necessity for thyroid hormone replacement therapy for a minimum of 6 months [57].

In a study of 137 patients with advanced NSCLC, of whom 99 were treated with nivolumab and 38 were treated with pembrolizumab, thyroid disorders were more frequent in patients with preexisting antithyroid antibodies compared to patients without preexisting antithyroid antibodies (20% vs. 1%, respectively; *p* < 0.001). A multivariate analysis revealed an independent association of preexisting antibodies with irAEs (odds ratio (OR), 3.25; 95% CI, 1.59–6.65; *p* = 0 .001 [97].

A prospective study of 26 patients with malignant diseases who received ICPi demonstrated an early significant increase (≤4 weeks) in the serum Tg levels and thyroid autoantibodies, anti-TgAbs and anti-TPOAbs, in the ir thyroid disorders group compared to the no ir thyroid disorders group (*p* < 0.05) [98].

In a retrospective observational study enrolling patients with advanced solid tumors who developed nivolumab-induced thyroid disorders with an incidence of 14%, positive anti-TgAbs at the baseline but not anti-TPOAbs were significantly associated with ir thyroid disorders (OR, 26.5; 95% CI, 8.18–85.8) [99].

A prospective study of 66 patients treated with nivolumab demonstrated a significantly higher prevalence of positive anti-TgAbs and/or anti-TPOAbs at the baseline in patients with destructive thyroiditis compared to patients without thyroiditis (three out of four vs. three out of 62, respectively; *p* = 0.002) [100].

Thyroid disorders related to pembrolizumab in NSCLC patients from KEYNOTE-001 (NCT01295827) were demonstrated to be strongly associated with antithyroid antibodies. Among the 51 patients treated, there were three patients with hypothyroidism and 48 patients with normal thyroid function at the baseline. Ir thyroid disorders requiring thyroid replacement occurred in 10 out of 48 (21%, 95% CI 10–35) patients. Antithyroid antibodies positivity was observed in eight out of 10 patients with ir thyroid disorders, as opposed to three out of 38 patients without ir thyroid disorders (80% vs. 8%, respectively; *p* < 0.0001) [101].

A retrospective study including 280 patients treated with nivolumab or pembrolizumab demonstrated a higher proportion of patients with high anti-TgAbs levels in the ir thyroid disorders group (*p* = 0.05) [102].

In a study of 93 patients with advanced cancer treated with at least one dose of pembrolizumab, showing an incidence rate of ir thyroid disorders of 14%, the positivity of anti-TPOAbs was observed in the minority of patients (four out of 13 (31%)). Three out of four patients with positive anti-TPOAbs had a preexisting diagnosis of hypothyroidism. The positivity of anti-TPOAbs at the baseline in two patients with pembrolizumab-induced thyroiditis was related to new-onset subclinical hypothyroidism in one patient and recurrent hypothyroidism requiring a >50% increase in L-thyroxine dose in the other patient. The negativity of anti-TPOAbs at the baseline was observed in 11 patients treated with pembrolizumab without thyroid impairment. The positivity of anti-TPOAbs at the baseline or in the history of hypothyroidism seemed to predispose patients towards the worsening or recurrence of hypothyroidism related to anti-PD-1 mAbs. Considering the absence of anti-TPOAbs in most patients with ir thyroiditis both during ir thyroid impairment and at the baseline, an antibody-independent mechanism or the presence of other thyroid autoantibodies not measured was postulated. Nevertheless, this study could not draw firm conclusions, given the small sample size and the absence of baseline anti-TPOAbs in the entire cohort [22].

In a retrospective cohort study enrolling 200 patients treated with nivolumab and revealing an incidence of ir thyroid disorders of 33.5%, anti-TPOAbs and anti-TgAbs were tested in 17 patients (8.5%) at the time of ir thyroid impairment. Five patients showed double positivity, five patients showed double negativity, and five patients showed positivity only for anti-TgAbs, while no patient showed positivity only for anti-TPOAbs. Interestingly, anti-TgAbs rather than anti-TPOAbs, which are the hallmarks of autoimmune thyroiditis, were related to overt ir thyroid disorders. Considering that five patients showed a double negativity for anti-TPOAbs and anti-TgAbs, it was hypothesized that other mechanisms, yet unexplored, underlie ir thyroid disorders [87].

Similarly, a nonconventional mechanism underlying ir thyroid disorders was insinuated by a retrospective analysis of the data of 24 Japanese patients with malignant melanoma (aged 17–85 years; 54% female), particularly in Th2-dominant patients. Ir thyroid disorders were observed in seven patients (29%), three of whom developed hypothyroidism with precedent thyrotoxicosis, while the remaining four patients developed hypothyroidism without precedent thyrotoxicosis. The antithyroid autoantibodies were evaluated in four patients, one of whom showed elevated titers. In this study, neither anti-TPOAbs or anti-TgAbs increased the risk for nivolumab-induced thyroid disorders [103].

In a retrospective review of patients treated with anti-PD-1 mAbs, either as monotherapy (pembrolizumab/nivolumab) or in combination with anti-CTLA-4 mAbs, in a single UK regional cancer center, no association between the clinical pattern of ir thyroid impairment (hyperthyroidism followed by hypothyroidism or de novo hypothyroidism) and the presence of thyroid autoantibodies was observed [104].

A low frequency of positive antithyroid autoantibodies was observed in a recent descriptive retrospective study analyzing 11 patients who received anti-PD-1 mAbs (nivolumab or pembrolizumab) and presented ir thyroid disorders. Anti-TgAbs and anti-TPOAbs were positive in 18% of patients. TRAbs were assessed in five patients with thyrotoxicosis: three patients with painless thyroiditis and two patients with primary hyperthyroidism, and were found negative [105].

To date, there is little evidence about the pathogenic role of TRAbs in ir Graves’ disease, as opposed to the ever-increasing clinical relevance of TRAbs in up to 95% of spontaneous Graves’ disease [106,107,108]. Either the negativity [65] or positivity [56,66,67,109] of TRAbs has been reported in anti-CTLA-4-induced Graves’ disease. In the setting of nivolumab-induced Graves’ disease, negative TRAbs had been described in three [69,110,111] case reports, while TRAbs has not been evaluated in another case report [112]. Recently, Kurihara et al. reported the development of Graves’ disease with positive TRAbs simultaneously with the development of type 1 diabetes related to nivolumab [113]. Additionally, Yamada et al. reported the first case of new-onset Graves’ disease related to nivolumab, confirmed by a diffusely increased thyroid uptake in scintigraphy and conversion of TRAbs from negative before the first administration of nivolumab to positive before the second administration of nivolumab [114].

Whether thyroid autoantibodies contribute to the pathogenesis of ir thyroid disorders or are “innocent bystanders” has yet to be clarified.

## 10. The Association of Ir Thyroid Disorders with ICPi Efficacy

A growing body of systematic reviews and meta-analyses [115,116,117,118,119] suggest that irAEs, including endocrine irAEs [116,119], could be designated as a predictive factor for the therapeutic efficacy of ICPi, being related to the improvement of the overall response [115,117,118], overall survival (OS) [115,116,117,118], and progression-free survival (PFS) [116,117,118] compared to the absence of irAEs. A stronger association of irAEs with response to anti-PD-1/anti-PD-L1 mAbs compared to the response to anti-CTLA-4 mAbs has been observed, likely due to intraclass differences in the indications, mechanisms, and time course of the treatments. Prospective well-powered studies are needed to analyze the impacts of the features of IrAEs, such as the site, severity, onset time, and management, on the efficacy of ICPi [29]. However, the association of irAEs with ICPi efficacy is not a consistent finding [120]. A detailed discussion of the association of irAEs with ICPi efficacy is beyond the scope of the present review.

Herein, we focus on the mounting data indicating an association of ir thyroid disorders with the therapeutic efficacy of ICPi [87,99,101,121,122,123], as depicted in Table 5.

In a study of 200 cancer patients treated with nivolumab, the group with ir thyroid disorders showed a significantly longer median OS than the group with no ir thyroid disorders in the total cohort (16.1 vs. 13.6 months, hazard ratio (HR) 0.61; 95% CI 0.39–0.93) and in the subgroup of lung cancer (not reached vs. 14.2 months, HR 0.51; 95% CI 0.27–0.92) but not in the subgroup of malignant melanoma (12.0 vs. 18.3 months, HR 1.54; 95% CI 0.67–3.43) [87]. Overt ir thyroid disorders were associated with a significantly longer median OS compared to no ir thyroid disorders (*p* = 0.033), but the subclinical ir thyroid disorders were not (*p* = 0.348) [87].

A study of 168 patients with advanced solid tumors treated with nivolumab showed a longer OS in patients with ir thyroid disorders compared to no ir thyroid disorders, but the result was not statistically significant (HR, 0.52; 95% CI, 0.25–1.11; *p* = 0.09) [99]. In another study, among 51 patients with advanced NSCLC who received pembrolizumab, the OS was significantly longer in patients with ir thyroid disorders (HR, 0.29; 95% CI 0.09–0.94; *p* = 0.04) [101].

A retrospective review of 174 patients who received nivolumab or pembrolizumab for metastatic or unresectable advanced cancer showed significantly longer median PFS (66 vs. 27 weeks, HR: 0.50, 95% CI: 0.26–0.89, *p* = 0.02) and OS (median: 156 vs. 59 weeks, HR: 0.34, 95% CI: 0.13–0.75, *p* = 0.01) in the ir thyroid disorders group vs. euthyroid group. Ir thyroid disorders were designated as an independent prognostic factor for the OS (HR: 0.42, 95% CI: 0.16–0.97, *p* = 0.04) [121].

In a study of 58 patients treated with nivolumab or pembrolizumab for metastatic NSCLC (stage IV), the ir thyroid disorders group compared to the euthyroid group showed significantly longer OS (median 118.0 days (73.0–267.0) vs. 71.0 days (28.0–160.0), log-rank *p* = 0.025) and PFS (18.0 days (73.0–267.0) vs. 61.0 days (28.0–130.0), log-rank *p* = 0.014) [122].

In a retrospective study of patients treated with nivolumab for NSCLC, after a median follow-up of 9 months (95% CI, 7.5–10.3), death in the ir thyroid disorders group was less frequent compared to the control group (6.7% vs. 33.3%), with a trend toward a higher OS in the ir thyroid disorders group (HR: 0.16 (95% CI, 0.02–1.15); *p* = 0.07) [123].

Patients with one irAE and multiple irAEs, including ir thyroid disorders, demonstrated incrementally improved OS compared to patients with no irAEs in multivariable models adjusting for the ICPi duration, according to a retrospective cohort study in five academic institutions worldwide enrolling 623 patients with stage III/IV NSCLC treated with anti-PD-(L)1 mAbs [78].

Nevertheless, many issues concerning the association of ir thyroid disorders with the efficacy of ICPi remain unresolved. Firstly, further large-scale prospective studies are warranted to validate the association of ir thyroid disorders with the therapeutic efficacy of ICPi. Secondly, whether the association of ir thyroid disorders with the therapeutic efficacy of ICPi is thyroid-specific has yet to be clarified. In fact, the impact of the cancer type on the relationship of irAEs with the therapeutic efficacy of ICPi is uncertain. The histological characteristics of the site of the tumor may influence the immune response, which, in turn, is implicated in the development of irAEs. On the other hand, the prevailing toxicity of ICPi appears to result from nonspecific effects on the immune system independently of the cancer type. Thirdly, the correlation of the severity of ir thyroid disorders with the therapeutic efficacy of ICPi is underexplored. So far, data concerning the association of the severity of irAEs with the therapeutic efficacy of ICPi are scarce and inconclusive. Grade ≥3 irAEs have been correlated with an improved objective response rate (ORR) (25% vs. 6%; *p* = 0.039) and extended median time to progression (30 vs. 10 weeks; *p* = 0.004) compared to the absence of grade ≥3 irAEs in patients with advanced cancer treated with ICPi [124]. However, low-grade irAEs are associated with a higher ORR (*p* = 0.017) and longer time to the next therapy or death (*p* = 0.008) in nonmelanoma patients treated with anti-PD-1 mAbs [125]. It has been postulated that severe irAEs can lead to considerable morbidity and often necessitate the administration of immunosuppressives [126], which may undermine the response to ICPi. More prospective studies are needed to investigate this hypothesis. Notably, in a recent systematic review and meta-analysis, grade 3 and 4 irAEs were associated with increased ORR but worse OS [117]. The first study that designated thyroid disorders related to anti-PD-1 mAbs as independent predictive factors for a favorable clinical outcome revealed a severity-dependent association of ir thyroid disorders with the objective response and durable disease control [121]. Finally, whether the relationship of irAEs with the therapeutic efficacy of ICPi is ascribed to a longer exposure of responders to ICPi, which potentially increases the risk for irAEs, has yet to be explored.

The identification of a causative link connecting the irAEs with the therapeutic efficacy of ICPi could strengthen this connection. Several relevant links are under evaluation, including: (i) increased T-cell responsiveness, which results in the production of proinflammatory cytokines by T cells and Treg deletion, (ii) the activation of B cells to produce antibodies against antigens shared by tumors and organs affected by irAEs, (iii) off-target effects on normal tissues expressing the targeted immune checkpoint ligand, (iv) the increased production of proinflammatory cytokines and chemokines from activated immune cells that results in organ-specific inflammation, (v) genetic susceptibility implicating HLA haplotypes, and (vi) environmental factors, including the microbiome [122].

Apart from ir thyroid disorders per se, the positivity of thyroid autoantibodies has also emerged as a potential predictor of favorable clinical outcomes. The association of preexisting thyroid autoantibodies with an improved median PFS compared to the absence of preexisting thyroid autoantibodies was observed in a study of 137 patients with advanced NSCLC treated with anti-PD-1 mAbs [97].

## 11. An Integrative Framework of the Association of ICPi with Ir Thyroid Disorders

The path forward for the illumination of the pathogenesis of ir thyroid disorders could be an integrative framework of the association of ICPi with ir thyroid disorders, suggested very recently by the inspiring systematic review of Zhan et al. The authors gathered all available data on the biological background discussed earlier in Section 3 of the present review, entitled “The biological background of ir thyroid disorders”, to conceptualize the framework of the association of the ICPi-induced immune response with the development of ir thyroid disorders. This framework was suggested to integrate T-cell-mediated immune responses, B-cell-mediated immune responses, specific cytokines, and genetic susceptibility [127].

Central in this framework is considered T-cell-mediated cellular immunity. In fact, anti-PD-1 mAbs have been shown to activate preexisting CD8^+^ T cells more often than anti-PD-L1 and anti-CTLA-4 mAbs, rationalizing the predilection of anti-PD-1 mAbs for the destruction of the thyroid. Ir Graves’ disease has been suggested as the initial autoimmune ir thyroid disorder, which triggers the exposure of thyroid cell antigens to immune cells, which, in turn, results in the development of ir autoimmune hypothyroidism due to the predominance of Th1 cells. Additionally, anti-PD-1 mAbs may stimulate thyroid autoimmunity via counteraction of the inhibitory effect of Treg on autoimmunity, given that PD-1 is critical for the proliferation and differentiation of Treg [127].

So far, no consensus on the contribution of the B-cell-mediated humoral autoimmune response to the pathogenesis of ir thyroid disorders exists. Both the negativity or positivity of thyroid autoantibodies has been described in the setting of ir thyroid disorders, while it has also been speculated that thyroid autoantibodies can be the result of humoral immune response triggered by the release of thyroid antigens in the context of destructive thyroiditis. Whether patients with previous subclinical autoimmune thyroid diseases are at an increased risk for ir thyroid disorders has yet to be clarified [127].

A suggested mechanism through which ICPi may confer a genetic susceptibility to thyroid autoimmunity is the upregulation of HLA-DR expression, as described in the setting of pembrolizumab-induced thyroiditis [127].

A speculative mechanism underlying anti-PD-1 mAbs-induced ir thyroid disorders is the polarization of the immune response towards a predominant Th1 profile, known to foster the genesis of organ-specific autoimmune diseases, including Hashimoto’s thyroiditis. This polarization is indicated by the integration of increased levels of proinflammatory Th1 cytokines (IFN-γ and IL-2), produced by an increased number of CD4^+^ Th1 cells with decreased Th2 cytokine activity [127].

Finally, aging and sex hormones have been speculated as components of the immunological background of ICPi-induced thyroid disorders [127].

## 12. Is There a Distinguishing Feature of Ir Thyroid Disorders in the Setting of Hematological Cancers Compared to Solid Tumors That Hampers the Appliance of Data Derived from the Latter in the Former?

A fundamental difference between ir thyroid disorders in the setting of hematological malignancies and those in the setting of solid tumors is that the latter concern the toxicity of various ICPi administered most often as the first-line treatment, while the former concerns mainly the toxicity of nivolumab and of pembrolizumab for patients with relapsed or refractory (r/r) classic Hodgkin’s lymphoma (cHL), the only FDA-approved ICPi indications in hematology so far [2,3,4,128].

It is conceivable that r/r cHL patients are heavily pretreated and have been subjected to hematopoietic stem cell transplantation (HSCT). HSCT is an aggressive antineoplastic therapeutic modality preceded by conditioning regimens consisting of high-dose antiblastic treatments associated or not with total body irradiation (TBI), resulting in complications that affect most systems and organs. The endocrine glands, including the thyroid, constitute one of the major targets of post-HSCT complications, because they contain a relatively high percentage of growing cells [129]. Thus, it is difficult to decide whether a thyroid disorder occurring during ICPi treatment is attributed to ICPi itself or to previous treatments.

Indeed, the spectrum of early and late endocrine post-HSCT complications is wide, ranging from subclinical to life-threatening adverse events. Several risk factors have been suggested, including underlying diseases; previous pretransplant therapies; a patient’s age at the time of HSCT; and TBI (use, cumulative dose, and administration schedule), as well as post-HSCT treatments [130].

Interesting information on endocrine post-HSCT complications was derived from the review of Orio et al., which indicated overt or subclinical thyroid disorders as the third-most common post-HSCT endocrinopathy observed in about 30% of HSCT recipients, following the impairment of the hypothalamic–pituitary–gonadal axis and the impairment of the hypothalamus–pituitary–adrenal axis observed, respectively, in two-thirds and 40–50% of HSCT [11]. Likewise, in a study of 259 patients with normal baseline thyroid function tests and at least 1-year survival after allogeneic HSCT, thyroid disorders—mainly hypothyroidism—have been reported as a common long-term complication after allogeneic HSCT, occurring in 25% of patients. The significant risk factors were older age, pretransplant active disease, and TBI [131].

The impact of HSCT on the H–P–T axis is both indirect and direct. The indirect impact is the development of functional hypothalamic hypothyroidism, known as NTIS or “low T3 syndrome”. NTIS is conceived as a key compensatory response of the pituitary—the so-called “endocrine master gland” [132]—to the nutritional/metabolic stresses ascribed to multiple adverse conditions—namely, the hematological neoplasm itself, conditioning regimens consisting of high-dose antiblastic treatments, and the administration of corticosteroids, especially in an autologous HSCT setting, as well as the onset of new diabetes, a rare but potentially severe complication of HSCT [130]. Albeit most often transient, NTIS is expected to persist for as long as the triggering adverse conditions persist. For instance, NTIS has been reported in about 30% of patients at 3 months after autologous HSCT, but it was recovered in all patients at 12 months. Expectedly, the incidence of hypothyroidism was higher in patients who had received neck/thoracic radiotherapy 15–36 months before HSCT compared to “the no neck/thoracic radiotherapy group” (50% vs. 1.3%, respectively). Although NTIS is usually asymptomatic, especially in autologous HSCT recipients, requiring no treatment, close monitoring until the normalization of the thyroid function tests is recommended.

The direct impact of HSCT on the H–P–T axis is multifactorial. First, the thyroid is one of the most sensitive organs to ionizing radiation, the latter being an integral part of the treatment of hematological malignancies. Radiation can impair the thyroid gland via interfering with the mitosis of thyroid cells, disrupting the blood supply to the thyroid, and triggering autoimmunity characterized by the increased expression of anti-TPO Abs and anti-Tg Abs. Beyond the well-established elevated risk for external beam radiotherapy (EBRT)-induced hypothyroidism in cHL survivors more than 25 years after exposure to EBRT [133], radiotherapy in the form of TBI can cause permanent or transient hypothyroidism. A higher incidence of TBI-induced hypothyroidism has been related to a single dose of TBI (46–48%) compared to fractionated TBI (15–16%). Alarmingly, the estimated 10-year cumulative incidence of thyroid nodules in ultrasound sonography in children after TBI hovers at 16%, with half of the cases being potentially malignant [134]. Syrjala KL et al. reported the development of hypothyroidism in 10–15% of patients who received TBI [135], and Kauppila, et al. demonstrated that hypothyroidism was more frequent with TBI doses >20 Gy [52].

Second, chemotherapy with [53] or without [54] radiotherapy administered for the treatment of cHL is often related to the development of thyroid disorders. Cima et al. reported a 35.7% prevalence of thyroid disorders in pediatric cHL patients—HSCT recipients who were subjected to chemotherapy-only conditioning, emphasizing the need for a long-term surveillance of the thyroid function and morphology in HSCT irrespective of the TBI. In this study, autologous HSCT was an independent risk factor for thyroid autoimmunity. The mean thyroid volume z-score was significantly lower in the autologous and the allogeneic HSCT groups compared to the control [136]. Studies comparing the standard chemotherapeutic combination regimen consisting of Adriamycin, Bleomycin, Vinblastine, and Dacarbazine (ABVD) with the previously used MOPP regimen (nitrogen mustard (Mechlorethamine), vincristine, procarbazine, and prednisone) [137] in terms of the thyroid toxicity are still lacking.

Third, the administration of iodinated contrast agents for imaging techniques can lead to hypothyroidism in predisposed individuals due to a defective escape from the Wolf–Chaikoff effect—a self-regulatory mechanism known to protect the patients against an overload of iodide [138]. For instance, an incidence of elevated TSH levels of 76.5% has been reported in patients with advanced Hodgkin’s disease following lymphography, persistent for a median period of 3 months [54].

Fourth, the derangement of the immune system within the first 6 months after autologous HSCT has been incriminated for the increased frequency of transient subclinical hyperthyroidism or hypothyroidism with a mild elevation of autoantibodies and, occasionally, with ultrasound evidence indicative of chronic thyroiditis (i.e., a nonhomogeneous hypoechoic pattern of the thyroid).

Fifth, ultrasound evidence indicative of chronic thyroiditis associated with normal thyroid function has been described 2–10 years after HSCT as an adverse effect of immunosuppressive therapies.

Taken together, in patients who receive ICPi after HSCT, distinguishing the thyroid toxicity of ICPi from the thyroid toxicity related to HSCT and the conditioning modalities is a daunting task. Additionally, it can be speculated that the risk of ir thyroid disorders may have an additive effect on the risk of thyroid disorders related to HSCT and the conditioning modalities. More light on this hypothesis could be shed by the comparison of the available data on ir hypothyroidism in the setting of r/r cHL with the preliminary data on the thyroid toxicity of nivolumab and pembrolizumab as frontline therapies of advanced cHL in phase II trials.

In clinical trials assessing the safety and efficacy of anti-PD-1 for r/r cHL, hypothyroidism has been related to nivolumab, with an incidence rate of 12% (Checkmate 205) [139,140,141] and with pembrolizumab with an incidence rate of 16% (Keynote-087) [142,143].

In brief, the results of a multicenter phase II PET-adapted study of sequential pembrolizumab monotherapy and AVD (doxorubicin, vinblastine, and dacarbazine) for untreated patients with cHL showed complete and near-complete responses in most patients, while pembrolizumab was related to the development of hypothyroidism or hyperthyroidism in three out of 30 patients [144]. The fact that this incidence of ir thyroid disorders was lower than that observed in heavily pretreated patients in the setting of Keynote-087 could insinuate a potential additive effect of the conditioning regimens on the development of thyroid disorders.

On the other hand, nivolumab administered in 51 patient aged 18 years or older with untreated advanced cHL (defined as III to IV and IIB with unfavorable risk factors) in Phase II CheckMate 205 has been related to development of hypothyroidism with an incidence rate of 14%, similar to that observed with the administration of nivolumab for r/r cHL [145]. These results, though inconclusive and difficult to explain, cannot be ignored and mandate further research.

## 13. Current Challenges and Future Perspectives

**Figure 3:**
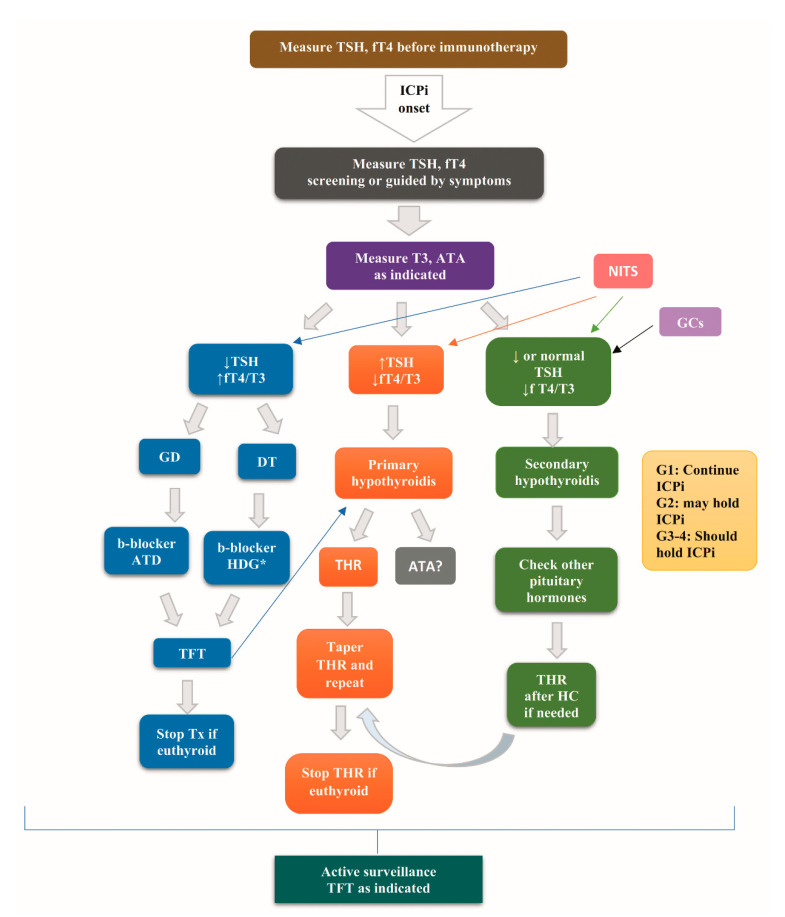
A comprehensive approach to ir thyroid disorders. * HDG are recommended for certain cases of grades 3 and 4 destructive thyroiditis. Abbreviations: ATA, antithyroid antibodies; ATD, antithyroid drugs; b-blockers; beta-blockers; DT, destructive thyroiditis; fT4, free T4; FU, follow-up; G, grade; GCs, glucocorticoids; HC, hydrocortisone; HDG, high-dose glucocorticoids; NTIS, nonthyroidal illness syndrome; TFT, thyroid function tests; THR, thyroid hormone replacement; TSH, thyroid-stimulating hormone; RAI, radioiodine; TT, total thyroidectomy; Tx, treatment.

A comprehensive approach to ir thyroid disorders is depicted in Figure 3. Such an approach poses many challenges.

With the growing amount of information concerning ir thyroid disorders, a multidisciplinary initiative should be directed at: (i) the development of a comprehensive list of terms of ir thyroid disorders; (ii) the development of a biospecimen repository for translational research; (iii) the standardization of algorithms to diagnose, treat, and record ir thyroid disorders; (iv) the aggregation and harmonization of data from big databases; and (v) the retrospective evaluation of electronic health records accessible to all involved healthcare providers. Such an initiative will help to better characterize the epidemiological profile of ir thyroid disorders and overcome the current barriers, including: (i) methodological differences among studies; (ii) differences in the policies of surveillance; (iii) the absence of universal recording strategies; and (iv) ICPi interclass and intraclass differences in thyroid immunotoxicity, likely reflecting distinct mechanisms of action, molecular structures, pharmacodynamics, and pharmacokinetics [146,147]. To facilitate the quantification of the risk of ir thyroid disorders, it is required to further assess the cumulative incidence of ir thyroid disorders, defined as the probability of occurrence over time in clinical trials [51]. Global registry strategies, leveraging artificial intelligence techniques and endorsing the new Side Effect Reporting Immuno-Oncology (SERIO) recommendations [148], herald a new era in immuno-oncology

In the era of precision medicine, the path forward for ir thyroid disorders is the individualization of the decision-making process. The identification and validation of tumor-specific, patient-specific, and ICPi class-specific patterns of susceptibility to ir thyroid disorders will help clinicians to stratify ICPi-treated patients according to the risk of ir thyroid disorders. For instance, the emerging predictive value of the pretreatment thyroid function status for the development of ir thyroid disorders, assessed by the TSH or free thyroxine levels, has yet to be determined [93,99,102].

Improved understanding of the biology of tumors, the biological background of ir thyroid disorders, the immune profiling of patients treated with ICPi, and the genetic background of thyroid autoimmune diseases and of cancer could lead to the validation of molecular biomarkers of ir thyroid disorders.

Given that the peripheral blood is readily accessible as opposed to tumor sampling, research has been focused on the identification of peripheral molecular predictive biomarkers. The integrated analysis of peripheral immunologic biomarkers at the cell, genomic, and epigenetic levels through cutting-edge technologies, including multicolor flow and mass cytometry, whole-transcriptome sequencing, epigenetic analysis, and multianalyte serum immunoassays, is expected to reveal predictive “signatures”. Such “signatures” should be further evaluated both retrospectively and prospectively in clinical trials to yield reproducible, sensitive, and specific biomarkers. To date, the validation of circulatory immunologic biomarkers in oncology has been hampered by assay variability, different platforms, and the lack of reference standards. These obstacles could be counteracted through the harmonization and standardization of key platforms [149].

Capturing irAE data pertinent to patient diversity will facilitate patient selection for immunotherapy. More well-designed clinical trials are needed to clarify ir thyroid impairment in the settings of preexisting cardiac disease, organ transplant, liver and kidney dysfunction, allogeneic stem cells transplant, and preexisting autoimmunity. The collaboration of leading experts in the corresponding specialties with the treating oncologists is required.

The identification of any causative link of thyroid autoantibodies with ir thyroid disorders could designate the thyroid autoantibodies as biomarkers of ir thyroid disorders. If the autoimmune nature of ir thyroid impairment is consolidated, some outstanding issues should be resolved, including: (i) the clarification of whether distinct thyroid autoantibodies have an equal impact on the development of ir thyroid disorders; (ii) the elucidation of whether ICPi precipitate underlying conventional autoimmune diseases or initiate a novel autoimmunity; (iii) the role of genetic factors, such as HLA haplotypes and genes polymorphisms, with emphasis on the genetical predisposition to polyglandular autoimmune syndromes, including ir thyroid disorders [92]; (iv) the flare of the baseline autoimmune disease observed in certain, but not all, ICPi-treated patients with preexisting autoimmunity [72,150,151]; and (v) the selection of patients with preexisting autoimmunity eligible for immunotherapy [151].

Strategies to separate antitumor immunity from immunotoxicity are currently under evaluation—namely, the development of bispecific or antibody-based alternative structures binding two distinct immunomodulatory targets and the administration of ICPi directly within the tumor microenvironment [16].

Additionally, the clinical relevance of biomarkers predicting not only the ICPi therapeutic efficacy but, also, the development of irAEs such as IL-6 and serum CTLA-4 should be further explored [152].

Immunosuppressive agents, which have been suggested for the treatment of irAEs, such as vedolizumab (anti-integrin α4β7), infliximab (a chimeric monoclonal anti-TNF-α antibody), tocilizumab (anti-IL6 receptor antibody), mycophenolate mofetil, cyclophosphamide, and intravenous immunoglobulins [153], merit further evaluation as a strategy to mitigate ir thyroid disorders.

Given that immunotherapy moves toward combinational regimens, including ICPi, further exploration of ir thyroid disorders in this setting is required. So far, the combination of different classes of ICPi has been shown to increase endocrine toxicity [30,31,43], though inconsistently [41], while combining ICPi with other anticancer agent results in variable endocrine toxicity [154,155,156,157,158,159,160,161].

The thyroid immunotoxicity related to the reintroduction of ICPi in patients for whom the withdrawal of ICPi was dictated by irAEs has yet to be defined. So far, a rechallenge poses ethical and scientific issues, given the reported recurrence of the same irAEs or the development of new irAEs in 28.8% [162] or 55% [163] of patients.

Most importantly, unraveling the determinants of the response to ICPi in hematological malignancies will open new avenues for not only the implementation of ICPi but, also, the mitigation of immune-related toxicities. Whether any tumor-intrinsic factors, such as the genetic landscape and/or the immunophenotype of hematological malignancies, or any extrinsic factors, such as preexisting autoimmunity or other comorbidities, determine a distinct profile of ir thyroid disorders in the setting of hematological malignancies has yet to be explored.

The current data on ir thyroid disorders in the setting of hematological malignancies are scarce and controversial [142,145,164,165,166,167,168]. Some representative data are depicted in Table 6.

Ir thyroid disorders are not a major concern in numerous studies addressing the efficacy and safety of ICPi in hematological malignancies [164,165,166,167]. On the contrary, other studies indicate hypothyroidism as a common irAE of pembrolizumab, with an incidence of 12.4% [142], and of nivolumab, with an incidence of 9% or 16%, implemented for the treatment of advanced/refractory HL [145,168]. This discrepancy might be ascribed to: (i) the heterogeneity of hematological malignancies; (ii) distinct molecular and genetic backgrounds; and (iii) differences in patient selection, study methodologies, and primary endpoints. These issues should be addressed in future studies. Additional issues to be considered in the design of future studies are the assessment of long-term outcomes, translational research, and the evaluation of the benefit–risk ratio.

**Figure 4:**
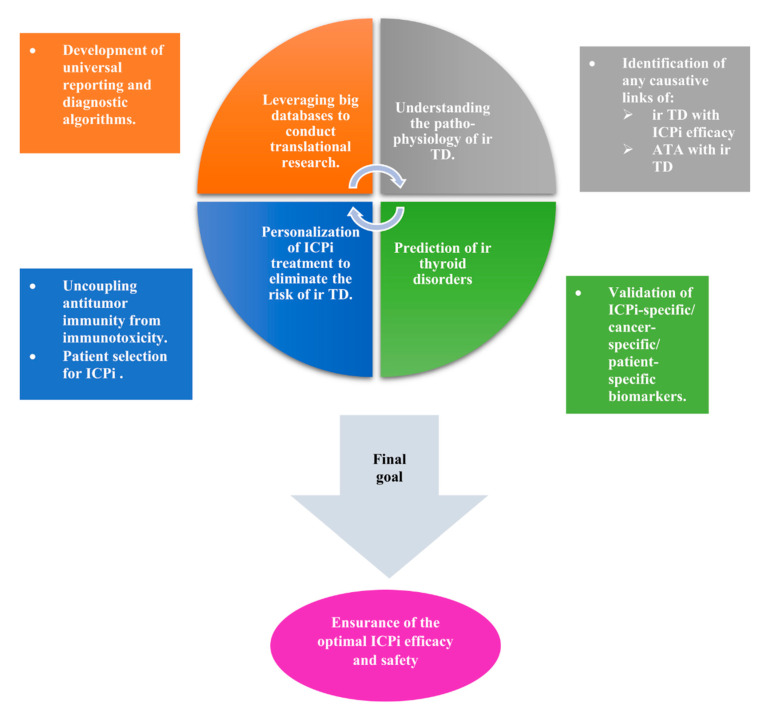
Future perspectives concerning ir thyroid disorders. The quarters of the inner circle depict the main future perspectives in the management of ir TD, and the corresponding rectangles depict the relevant initiatives. The final goal is to ensure optimal ICPi efficacy and safety. Abbreviations: ATA, antithyroid antibodies; ICPi, immune checkpoint inhibitors; TD, thyroid disorders.

Overall, Figure 4 recapitulates the main future perspectives concerning ir thyroid disorders.

With the emergence of novel ICPi-targeting checkpoint inhibitors other than PD-1/PD-L1 and CTLA-4 for the treatment of hematological malignancies, the landscape of ir thyroid immunotoxicity is expected to evolve rapidly [2,3].

Finally, in the era of the COVID-19 pandemic, the interrelationship of thyroid impairment with COVID-19 infection [169], which can be life-threatening, imposes the prompt diagnosis and management of ir thyroid disorders.

## 14. Conclusions

Until more data are available to clarify ir thyroid disorders in the setting of hematological malignancies, clinicians leverage the relevant data derived from solid tumors. Albeit reminiscent of conventional autoimmune thyroid diseases, ir thyroid disorders represent a novel entity, standing at the crossroads of oncology, immunology, cancer biology, and endocrinology. Although ir thyroid disorders are generally considered well-tolerated and manageable, many issues remain unresolved, including the biological background, the predictive biomarkers, and the prognostic value of ir thyroid disorders, as well as the ir thyroid toxicity in hematological malignancies. The appropriate management of immunotoxicity—in this case, of thyroid immunotoxicity—is a prerequisite for optimizing both the therapeutic efficacy of ICPi and the quality of life. An intimate interrelationship between oncologists, endocrinologists, and well-informed patients will provide optimal patient care.

## Figures and Tables

**Table 1:** Frequency of ir thyroid disorders according to the recent meta-analyses.

Ref. Enrolled Studies/ Type of Studies Ca Type (N * = Number of Studies; N ** = Number of Patients)	ICPi	Hypothyroidism	Hyperthyroidism
[26] 35 trials addressing irAEs in advanced melanoma, involving 6331 patients. Systematic Review and Meta-analysis Advanced Melanoma (N * = 35, N ** = 6331)	IPI (N * = 21, N ** = 2726) NIVO (N * = 7, N ** = 1017) PEMBRO (N * = 5, N ** = 1632)	IPI: 2.84 (1.46–4.57) NIVO: 7.02 (4.37–10.19) PEMBRO: 8.34 (7.01–9.77) PEMBRO + IPI: 16.34 (11.32–23.01) NIVO + IPI: 16.39 (13.50–19.490 IPI then NIVO: 11.22 (0.03–33.98) NIVO then IPI: 2.06 (13.85 –33.260	IPI: 0.90 (0.14–2.16) NIVO: 3.01 (1.96–4.24) PEMBRO: 3.34 (1.60–5.61) PEMBRO + IPI: 11.11 (7.05–17.07) NIVO + IPI: 10.16 (95.94–15.28) IPI then NIVO: 0.00 (0.00–10.43) NIVO then IPI: NR
[27] 9 RCTs addressing irAEs in advanced melanoma, involving 5051 patients. Systematic review and network meta-analysis Advanced melanoma (N * = 9, N ** = 5051)	IPI PEMBRO NIVO	IPI 3 mg/kg every 3 weeks was the treatment regimen with the lowest probability to cause hypothyroidism (median rank, 2; 95% CrI, 1–5) ^a^. NIVO 3 mg/kg every 2 weeks was the treatment regimen with the lowest risk of severe hypothyroidism (median rank, 3; 95% CrI, 1–7) ^a^.	NS
[28] 13 studies addressing anti-PD-1/anti-PD-L1 toxicity, involving 6676 patients. Systematic Review and meta-analysis Metastatic non-small cell lung cancer (N * = 7) Melanoma (N * = 3) Renal Cell Carcinoma (N * = 1) Bladder cell carcinoma (N * = 1) Head and Neck squamous cell carcinoma (N * = 1)	NIVO (N * = 6, N ** = 1534) PEMBRO (N * = 5, N ** = 1518 +/−) ATE (N * = 2, N ** = 751)	Among patients exposed to anti-PD-1 drugs: 214/3803 developed hypothyroidism (5.6%). In the control group: 24/3353 developed hypothyroidism (0.7%) ⮚ ATE: All grades: 1.1 (0.5–2.1) Serious: 0.1 (0.0–0.7) ⮚ NIVO: All grades: 5.9 (4.7–7.2) Serious: 0.2 (0.0–0.6) ⮚ PEMBRO: All grades: 7.6 (6.4–9.1) Severe: 0.1 (0.0–0.5) ⮚ TOTAL: All grades: 5.6 (4.9–6.4) Serious: 0.2 (0.1 to 0.3) ⮚ In the meta-analysis, patients treated with anti-PD-1 drugs were at a higher risk for any grade hypothyroidism (OR, 6.92, 3.25–14.75, *p* < 0.001) compared to controls who received standard treatment (chemotherapy, targeted drugs, or both).	NS
[29] 11 studies addressing anti-CTLA-4 toxicity, involving 7088 patients. Systematic Review and meta-analysis Melanoma (N * = 4, N ** = 2862) Metastatic non-small cell lung cancer (N * = 2, N ** = 1216) Small cell lung cancer (N * = 2, N ** = 1126) Metastatic castration-resistant prostate cancer(N * = 2, N ** = 799) Mesothelioma (N * = 1, N ** = 569)	IPI (N * = 9, N ** = 3280) TREME (N * = 2, N ** = 705)	IPI: All grades: 2.5 (2.0–3.1) Serious: 0.3 (0.2–0.6) TREME: All grades: 2.7 (1.6–4.2) Serious: 0.6 (0.2–1.4) Total: All grades: 2.5 (2.0–3.0) Serious: 0.4 (0.2–0.6) ⮚ Higher risk of hypothyroidism (OR, 7.86; 95% CI, 4.10–15.04) in the intervention arms (IPI or TREME) compared to the control group (placebo, chemotherapy, radiation therapy, or vaccine). ⮚ Same trends for severe irAEs	IPI: All grades: 0.3 (0.1–0.5) Serious:0.3 (0.1–0.5) TREME: All grades: 0.0 (0.0–0.5) Serious:0.0 (0.0–0.5) Total: All (CTCAE) grades: 0.2 (0.1–0.4) Serious: 0.2 (0.1–0.4) ⮚ No higher risk of hyperthyroidism (OR 3.78, 95% CI 0.94–15.17) in the intervention arms (IPI or TREME) compared to the control group (placebo, chemotherapy, radiation therapy, or vaccine). ⮚ Same trends for severe AEs.
[30] 101 studies addressing endocrine irAEs, involving 19,922 patients. Systematic Review and meta-analysis Melanoma (N * = 69/152) Non-small cell lung carcinoma (N * = 31/152) Renal cell carcinoma (N * = 11/152)	IPI (N ** = 4430) TREME (N ** = 1171) NIVO (N ** = 3317) PEMBRO (N ** = 4485) ATE (N ** = 998) AVE (N ** = 316) DUVRA (N ** = 191)	IPI: 3.8 (2.6–5.5) ATE: 6.0 (4.2–8.4) DUVRA: 4.7 (2.5–8.8) NIVO: 8.0 (6.4–9.8) PEMBRO: 8.5 (7.5–9.7) AVE: 5.5 (3,5–8.7) DURVA + TREME: 10.2 (5.6–17.9) IPI + PEMBRO: 15.1 (10.6–21.8) IPI + NIVO: 16.4 (11.7–22.5)	IPI: 1.4 (0.8–2.4) NIVO: 2.8 (2.1–3.8) PEMBRO: 3.7 (2.8–4.7) AVE: 2.3 (0.6–8.6) IPI + NIVO: 9.4 (7.1–12.3) IPI + PEMBRO: 10.4 (6.6–16.1)
[31] 38 RCT addressing irAEs in advanced solid tumors, involving 7551 patients. Systematic Review and meta-analysis Melanoma (N * = 25, N ** = 3346) Non-small cell lung cancer (N * = 10, N ** = 1906) Renal cell carcinoma (N * = 6, N ** = 664)	NIVO (N ** = 2494) PEMBRO (N ** = 2459) IPI (N ** = 1013) ATE (N ** = 4953)	Overall incidence: 6.6 (5.5–7.8) Predicted incidence: IPI: 3.8 (1.9–7.8) Combination: 13.2 (6.9–23.8).	Overall incidence: 2.9 (2.4–3.7) Predicted incidence: anti-PD-L1: 0.6 (0.2–1.8) Combination: 8.0 (4.1‒15.3)
[32] 11 RCTs addressing irAEs of ICPi combination, involving 5307 patients. Meta-analysis Advanced/Metastatic melanoma (N * = 6, N ** = 2194) Non-small cell lung cancer (N * = 1) Small-cell lung cancer (N * = 1) Recurrent glioblastoma (N * = 1, N ** = 40) Advanced renal-cell carcinoma (N * = 1, N ** = 1096) Recurrent metastatic sarcoma (N * = 1, N ** = 85)	NIVO (N * = 11, N ** = 3467) IPI (N * = 11, N ** = 2976)	Combination: RR for all-grade hypothyroidism: 1.71 (95% CI, 1.38–2.13; *p* < 0.00001)	Combination: RR for all-grade hyperthyroidism: 2.84 (95% CI, 1.71–4.72, *p* < 0.0001
[33] 21 trials addressing irAEs, involving 11,454 patients. Meta-analysis Non-small cell lung cancer (N * = 8) Melanoma (N * = 6) Small cell lung cancer (N ** = 2) Prostate cancer (N ** = 2) Renal cell carcinoma (N * = 1) Bladder cancer (N * = 1) Squamous cell cancer of the head and neck (N * = 1)	IPI (N ** = 272) NIVO (N ** = 1534) PEMBRO (N ** = 1522) ATE (N ** = 751)	All ICPi: All grades: 5.1 (3.8–6.8) High grade: 0.3 (0.2–0.5) Pooled RR for all grade: 6.81 (4.20–11) *p* < 0.001 Pooled RR for high grade: 1.15 (0.44–3.05) anti-PD1/anti-PD-L1: RR: 8.05 (4.26–15.2) anti-CTLA-4: RR: 2.02 (0.39–10.5)	NS
[34] 10 clinical trials addressing irAEs, involving 5, 291 patients. Meta-analysis Malignant melanoma (N ** = 3868) Castrate-resistant prostate cancer (N ** = 789) Non-small cell lung cancer (N ** = 463)	IPI (N * = 5) NIVO (N * = 3) TREME (N * = 1) PEMBRO (N * = 1)	1.6–8.9 RR for all grades: 8.26 (95% CI: 4.67–14.62 *p* < 0.00001)	0.4–3.5 RR for all grades: 5.48 (95% CI: 1.33–22.53; *p* = 0.02)
[35] 10 studies addressing irAEs, including 8 RCTs involving 2716 patients. Meta-analysis Lung cancer (N * = 2) Melanoma (N * = 3) Metastatic sarcoma (N * = 1) Urothelial carcinoma (N * = 1) Recurrent glioblastoma (N * = 1)	NIVO (N ** = 2359) IPI (N ** = 1758)	⮚ Hypothyroidism any-grade ranks second among irAEs related to ICPi combinations. ⮚ Incidence of any-grade hypothyroidism in the ICPi combination vs. ICPi monotherapy group: 13.8 (194/1401 cases) vs. 7.2 (95/1315 cases).	⮚ Hyperthyroidism any-grade ranks fifth among irAEs related to ICPi combinations. ⮚ Incidence of any-grade hyperthyroidism in the ICPi combination vs. ICPi monotherapy group:

Values are percentages of incidence (95% confidence intervals) unless cited otherwise. All relative risks (RR) refer to comparison of ICPi arms to non-ICPi arms. N * = number of studies; N ** = number of patients. ^a^: Ranking of the possibility of being the ICPi regimen with the lowest risk to cause ir thyroid disorders was based on an estimation of the median (95% CI) of the posterior distribution for the rank of each studied ICPi regimen. This analysis indicated IPI 3 mg/kg every 3 weeks as the treatment regiment associated with the lowest risk for any hypothyroidism (median rank, 2; 95% CI, 1–5) compared to: chemotherapy, ipilimumab 10 mg/kg every 3 weeks, pembrolizumab 10 mg/kg, every 2 weeks, nivolumab 1 mg/kg every 3 weeks and ipilimumab 3 mg/kg every 3 weeks, nivolumab 3 mg/kg every 2 weeks, pembrolizumab 10 mg/kg every 3 weeks, and pembrolizumab 2 mg/kg every 3 weeks. Abbreviations:.anti-CTLA-4, antibodies against cytotoxic T-lymphocyte antigen 4, anti-PD-1, antibodies against programmed cell death protein-1; anti-PD-L1, antibodies against PD-1 ligand molecule; ATE, atezolizumab; AVE, avelumab; Ca, cancer type; CI, confidence interval; CrI, credible interval; DUVRA, durvalumab; ICPi, immune checkpoint inhibitors; IPI, ipilimumab; N, number; NIVO, nivolumab; NS, not studied; OR, odds ratio; PEMBRO, pembrolizumab; RCT, randomized controlled trials; Ref, reference; RR, relative risk; TREME, tremelimumab; vs., versus.

**Table 2:** Frequency of ir thyroid disorders according to recent reviews.

	Ir Hypothyroidism			Ir Hyperthyroidism		
Ref.	Anti-CTL-A4	Anti-PD-1/PDL-1	Combo or Sequential Regimens	Anti-CTL-A4	Anti-PD-1/PDL-1	Combo or Sequential Regimens
[36]	2.5–5.2	3.9–8.5	10.2–16.4	0.2–1.7	0.6–3.7	8.0–11.1
[38]	2/29 cases	16/29 cases	11/29 cases (9/29 cases for sequential 2/29 for combo)	IPI: 4/6 cases	NIVO: 1/6 cases	IPI + anti-PD-1/anti-PDL-1: 1/6 cases
[39]	IPI: 6	NS	IPI + NIVO: 22	NS	NS	NS
[40]	TREME: 2.3	5.9	13.9	TREME: 2.6	3.3	8
[41]	4.3–11.0 ^a^ 5.2–5.9 ^b^	5.9	22 ^a^ 17 ^b^	2	1–4.7	10
[42]	IPI: Any G: 5 G 3–4: 0	NIVO: Any G: 11 G 3–4: 0	NIVO + IPI: Any G: 17 G 3–4: <1	IPI: Any G: 1 G 3–4: 0	NIVO: Any G: 4 G 3–4: 0	NIVO + IPI: Any G: 11 G 3–4: 1
[43]	NS	PEM melanoma: 8.7 NIVO melanoma: 8.6 NIVO SC- NSCLC: 4 NIVO NS- NSCLC: 6.6	NS	NS	PEM melanoma: 3.2 NIVO melanoma: 4.2 NIVO NS- NSCLC:1.4	NIVO + IPI: 9.9
[44]	IPI: 1.5–6.8 TREME: 2.3	NIVO: 9–10.8 PEM: 7–9.1 AVE: 5 ATE: 2.5–4.2 DUVRA: 5.5–9.6	NIVO + IPI: 4–27 PEM + IPI: 6–13.6 DUVRA + TREME: 5.9	IPI: 4 TREME: 0–3	NIVO: 2.7 PEM: 3.4–7.8 AVE: 0.4 ATE: 0.6–1.1 DUVRA: 4.9–5.7	NIVO + IPI: 4.3–14 PEM + IPI: 4.5–6 DUVRA + TREME: NR
[45]	IPI: 1.5–13.3 TREME: 2–3	Anti-PD-1: 2–3 Anti-PD-L1: 3	NS	TREME: <1–2.5	Anti-PD-1: <1	NS
[37]	Any ir thyroid disorder					
	Anti-CTLA-4		Anti-PD-1/anti-PDL-1		Combo or sequential	
	IPI: 4.7 (range 2.0–10.4)		ATE: 22.2 NIVO: 8.8 (range 2.0–10.4) melanoma: 11.4 other solid organ cancers: 5.5 PEMBRO: 15.6 (range: 10.6–27.4)		IPI + NIVO: 16.0 melanoma (24.9) lung cancer (11.6) renal cell cancer (15.5) IPI then NIVO: 15.1	

All percentages represent incidence (95% confidence intervals), except the percentages in [41] that represent the prevalence. ^a^ Secondary hypothyroidism. ^b^ Primary hypothyroidism. Abbreviations: anti-CTLA-4, antibodies against cytotoxic T-lymphocyte antigen 4, anti-PD-1, antibodies against programmed cell death protein-1; anti-PD-L1, antibodies against PD-1 ligand molecule; ATE, atezolizumab; AVE, avelumab; DUVRA, durvalumab; IPI, ipilimumab; NIVO, nivolumab; NR, not reported; NS, not studied; NS-NSCLC, non-squamous non-small-cell lung cancer; Ref, reference; SC-NSCLC, squamous-cell non-small-cell lung cancer.

**Table 3:** Key messages from pharmacovigilance studies regarding ir thyroid disorders.

Ref.	Type of Study/Methods	Key Messages
[5]	Review and critical appraisal of 30 pharmacovigilance studies addressing irAEs as of 25 February 2020 using both a disproportionality and descriptive approach. The aim of the review was to provide a global perspective for the management of irAEs in clinical practice.	⮚ Endocrinopathies rank first among other irAEs, with thyroid disorders being the most common endocrine irAE. ⮚ Relative frequencies: Endocrinopathies: 8.6% All thyroid disorders: 3.8% Hypothyroidism: 1.8% Hyperthyroidism: 1.5% ⮚ The lower than expected reporting registered for hypothyroidism (compared to RCTs) may be ascribed to report of multiple irAEs in the same patient. ⮚ Median time of onset of thyroid disorders related to anti-PD-1/PD-L1 mAbs: 92 days. ⮚ Thyroid disorders were preferentially recorded with anti-PD-1/PD-L1 mAbs.
[7]	Disproportionality analysis of signals of irAEs. Data source: the FAERS database from the respective FDA approval dates for each specific drug^a^ through 2017 Q2. Evaluation of signals of disproportionality reporting using the pharmacovigilance index reporting odds ratio (ROR) with 95% CI.	⮚ Thyroid disorders: NIVO: N = 289; ROR, **18.36** (95% CI, 16.25–20.73) PEMBRO: N = 52; ROR, **10.11** (95% CI, 7.66–13.34) IPI: N = 166; ROR, 7.76 (95% CI, 6.64–9.06) IPI + NIVO: N = 50; ROR, **26.89** (95% CI, 20.143–5.91) ⮚ Higher association of thyroid disorders with NIVO or PEMBRO compared to IPI monotherapy. ⮚ Association of combination of IPI plus NIVO with higher risk of thyroid dysfunction than either agent alone. ⮚ Signal strength: Hypothyroidism: NIVO: N = 160; ROR, **41.85** (95% CI, 35.38–49.51) PEMBRO: N = 27; ROR, **20.34** (95% CI, 13.86–29.87) IPI: N = 56; ROR, 8.16 (95% CI, 6.26–10.64) IPI plus NIVO: N = 20; ROR, **40.34** (95% CI, 25.77–63.16) Hyperthyroidism: NIVO: N = 50; ROR, **16.83** (95% CI, 12.64–22.42) PEMBRO: N = 7; ROR, 7.36 (95% CI, 3.49–15.50) IPI: N = 29; ROR, 8.74 (95% CI, 6.05–12.62) IPI plus NIVO: N = 19 cases, ROR, **54.61** (95% CI, 34.45–86.59) Thyroiditis: NIVO: N = 16; ROR, **30.22** (95% CI, 18.03–50.6) PEMBRO: N = 3; ROR, 17.39 (95% CI, 5.54–54.53) IPI: N = 14; ROR, 25.42 (95% CI, 14.87–43.44) IPI plus NIVO: N = 10; ROR, **159.50** (95% CI 83.76–303.75) TSH decreased: NIVO: N = 14; ROR:, 29.89 (95% CI, 17.21–51.90) PEMBRO: N = 4; ROR, 26.47 (95% CI, 9.79–71.57) IPI: N = 26; ROR, **39.18** (95% CI, 26.31–58.33) IPI plus NIVO: N = 2 TSH increased NIVO: N = 36; ROR, **29.06** (95% CI, 20.60–41.01). PEMBRO: N = 3; ROR, 7.28 (95% CI, 2.3–22.69). IPI: N = 16; ROR, 10.30 (95% CI, 6.28–16.90). IPI plus NIVO: N = 6; ROR, 38.81 (95% CI, 17.27–87.25).
[46]	Retrospective disproportionality analysis of FAERS database from 2014 Q1 through 2019 Q1. Disproportionality was calculated by the informataion component (IC) or ROR with full database as comparator, and only ROR when comparing different drug strategies. A signal was considered significant if lower limit of the 95% confidence interval (ROR_025_) > 1, with at least 3 cases. Threshold for statistical signal detection IC_025_ > 0 (IC_025:_ lower end of a 95% confidence interval for the IC).	⮚ Hypothyroidism (N = 885, 14.14%) ranks 1st and hyperthyroidism (N = 472, 7.54%) ranks 4th among endocrine irAEs. ⮚ Stronger association of hypothyroidism and hyperthyroidism with anti-PD-1 mAbs and combination of NIVO plus IPI regimen. ⮚ Most endocrine irAEs were related to anti-PD-1 mAbs (N = 3398, 54.28%), corresponding to IC_025_, 2.20 and ROR_025,_ 4.82. ⮚ Anti-CTLA-4 mAbs were responsible for a small proportion (N = 708, 11.31%) but stronger signal values (IC_025_, 2.84, ROR_025_, 7.68). ⮚ IPI showed the strongest signal of IPi-associated endocrine irAEs (IC_025_ = 2.84, ROR_025_, 7.69). ⮚ Higher association of hypothyroidism/hyperthyroidism with NIVO or PEMBRO compared to IPI monotherapy. ⮚ Significant lower reporting frequencies of hypothyroidism (ROR, 0.68; 95%CI, 0.59–0.78) and hyperthyroidism (ROR, 0.77; 95%CI, 0.63–0.93) for males compared to females. ⮚ Hypothyroidism showed higher reporting frequency than hyperthyroidism (885 vs. 472).
[47]	Observational, retrospective, and disproportionality analysis based on the VigiBase database, reporting suspected ir thyroid disorders from 1 January 2011 to 6 March 2019.	⮚ Most reports are related to lung cancer and melanoma. ⮚ Male predominance of ir thyroid disorders. ⮚ Over-report for the full database of: hypothyroidism (1125 reports for ICPi vs. 12495 for all drugs; IC, 4.28 (95% CI, 4.18–4.35) hyperthyroidism (926 reports vs. 7538 reports; IC, 4.66 (95% CI, 4.55–4.74) thyroiditis: 294 reports vs. 1237 reports; IC, 5.40 (95% CI, 5.21–5.54) thyrotoxic crisis: 11 reports vs. 288 reports; IC, 3.55 (95% CI, 2.61–4.20) ⮚ Over-report for patients treated with ICPi combination vs. ICI monotherapy for: hypothyroidism: ROR, 1.3 (95% CI, 1.1–1.7) hyperthyroidism: ROR, 1.9 (95% CI, 1.5–2.4) thyroiditis: ROR, 3.3 (95% CI, 2.3–4.8) thyrotoxic crisis: ROR, 11.5 (95% CI, 2.4–53.8) ⮚ All 11 thyrotoxic crisis cases occurred in malignant melanoma patients, of which seven were related to ICPi combination. ⮚ Anti-PD monotherapy vs. anti-CTLA-4 monotherapy showed over-report of: hypothyroidism: ROR, 2.4 (95% CI, 1.8–3.1) hyperthyroidism: ROR, 3.6 (95% CI, 2.5–5.3) thyroiditis: ROR, 1.9 (95% CI, 1.1–3.2) ⮚ NIVO was associated with most cases of: hypothyroidism (578 cases, 51.38%), hyperthyroidism (447 cases, 48.27%) thyroiditis (128 cases, 43.54%) ⮚ Time to onset of hypothyroidism significantly earlier with ICPi combination: anti-PD: 92 days (IQR: 53–146) anti-CTLA-4: 63 days (IQR: 42–70) ICPi combination: 46 days (IQR: 23–87) ⮚ Media duration of thyrotoxicosis: 35.5 days (IQR: 9–62.55). ⮚ Recovery or remission after standard treatment in most cases. ⮚ 10 cases (0.68%) resulted in major or fatal consequences, including 6 cases of hypothyroidism, 2 cases of thyrotoxic crisis, 1 case of hyperthyroidism and 1 case of thyroiditis. ⮚ Recovery in 59.81% of hypothyroidism, 78.35% of hyperthyroidism, and 75.16% of thyroiditis patients (comparison between hypothyroidism and hyperthyroidism, *p* < 0.0001; comparison between hypothyroidism and thyroiditis, *p* = 0.002). ⮚ Most reports came from patients with lung cancer and melanoma. ⮚ Male predominance of ir thyroid disorders.

^a^: Data on nivolumab (22 December 2014), pembrolizumab (4 September 2014), ipilimumab (25 March 2011), and nivolumab plus ipilimumab (25 March 2011) were analyzed from their respective FDA approval dates for each specific drug through 2017 Q2. Statistically greater RORs (i.e., ROR > 2, which means that the odds for this adverse event when using this drug is at least twice as much as for all the other drugs) are highlighted in bold. Abbreviations: anti-CTL-A4, anti-cytotoxic T-lymphocyte antigen 4; anti-PD-1, anti-programmed cell death 1; anti-PD-L1, anti-programmed cell death ligand 1; CI, confidence interval; FAERS, FDA Adverse Event Reporting System; IC, information component; ICPi, immune checkpoint inhibitors; IQR, interquartile range; IPI, ipilimumab; irAE (s), immune-related adverse event(s); mAbs, monoclonal antibodies; N, number of patients; NIVO, nivolumab; PEMBRO, pembrolizumab; Q, quarter; Ref, reference; RCTs, randomized controlled trials; ROR, reporting odds ratios.

**Table 4:** Guidelines for the treatment of ir thyroid disorders.

Ir Thyroid Disorder	Clinical Practice Guidelines Experts’ Committees [Ref]			
	ASCO [80]	SITC [89]	NCCN [90]	ESMO [91]
Hypothyroidism	G 1: Continue ICPi. with frequent TFT. G 2: May hold ICPi until resolution of symptoms. Endocrine consultation. TSH > 10 mIU/L or TSH > 4 mIU/L plus symptoms: THR. TFT Q 6–8 wk for THR titration until TSH normalization and, accordingly, annually or guided by symptoms. G 3–4: THR Hold ICPi. Endocrine consultation. IV L-thyroxine for myxedema.	G ≤ 2: L-thyroxine: 1.6 μg/kg/d (young, healthy) 25–50 μg (elderly, patients with CVD). TFT Q 6–8 wk for titration. Increments of L-thyroxine dose by 12.5–25 μg if indicated. After TSH normalization, TFT Q 1 y, or earlier if needed. G ≥ 3: Hold ICPi THR as G ≤ 2.	SH: Continue ICPi TFT TSH > 10 and/or symptoms: Start L-thyroxine Continue ICPi if no symptoms Endocrine consultation TFT Q 4–6 wk	THR (L-thyroxine: 50–100 μg/day.) SH: THR if fatigue. Τitration of L-thyroxine until TSH normalization. Inflammatory thyroiditis: prednisone orally 1 mg/kg tapered gradually. Consider holding ICPi if patient is symptomatic.
Thyrotoxicosis	G 1: Continue ICPi with frequent TFT. G 2: May hold ICPi until resolution of symptoms. Administer b- blockers. Hydration and supportive care. Thyrotoxicosis >6 wk, or clinical suspicion of GD: Diagnostic work-up for GD. Treat GD as indicated, preferably starting with thionamide. G 3–4: As in G2. For severe symptoms: Inpatient management. 1 to 2 mg/kg/d prednisone or equivalent tapered over 1 to 2 wk. Saturated solution of potassium iodide or thionamide.	b-blockers (e.g., atenolol 25–50 mg daily, titrate for HR < 90 if BP allows). TFT (mainly f T4) Q 2 wk Treat GD per standard guidelines. Hold ICPi if G ≥ 3.	No symptoms: Continue ICPi Administer b-blockers (propranolol or atenolol or metoprolol). TFT in 4–6 weeks: If normal: no further therapy. If abnormal: Diff dx of GD from destructive thyroiditis. If thyrotoxicosis evolves to hypothyroidism: Initiate L-thyroxine when TSH >10 mIU/L.	Administer b-blockers (propranolol or atenolol) and rarely carbimazole or steroids. Hold ICPi until resolution of symptoms.

Abbreviations: ASCO, American Society of Clinical Oncology; BP, blood pressure; CVD, cardiovascular disease; Diff dx, differential diagnosis; fT4, free T4; G, grade; GD, Graves’ disease; HR, heart rate; ICPi, immune checkpoint inhibitors; IV, intravenously; NCCN, National Comprehensive Cancer Network; Q, every, SH, subclinical hypothyroidism; SITC, Society for Immunotherapy of Cancer; TFT, thyroid function tests; THR, thyroid hormone replacement; TSH, thyroid stimulating hormone; wk, week; y, year. The treatment of ir hypothyroidism consists of the administration of L-thyroxine in all patients with TSH *>* 10 mIU/l [80] and in selected patients with 4 mIU/l *<* TSH *<* 10 mIU/l [92] and normal fT4 levels who present symptoms of hypothyroidism. The usual initiating dose of L-thyroxine is 1.6 μg/kg/day or lower (25–50 μg/day) for elderly or fragile patients with multiple comorbidities [80]. Titration is based on monitoring the TSH and fT4 levels every 6–8 weeks, according to the ASCO [80] and Society for Immunotherapy of Cancer (SITC) [89] guidelines, or every 4–6 weeks, according to the National Comprehensive Cancer Network (NCCN) guidelines [90]. Notably, any deficiency of cortisol should be corrected before the initiation of L-thyroxine [80].

**Table 5:** Representative studies addressing the association of ir thyroid disorders with ICPi efficacy.

Ref.	Study Patients	Results
[87]	200 patients treated with nivolumab at Kyoto University Hospital from 1 September 2014 to 31 August 2017.	⮚ Incidence of ir thyroid disorders: 33.5%. ⮚ Correlation of pre-treatment thyroid FDG-PET uptake with high incidence of overt ir thyroid disorders (adjusted OR,14.48; 95% CI, 3.12–67.19), but not with subclinical ir thyroid disorders. ⮚ OS in ir thyroid disorders group vs. no ir thyroid disorders group: Total cohort: 16.1 vs. 13.6 months (HR, 0.61; 95% CI, 0.39–0.93). Lung cancer: not reached vs. 14.2 months (HR, 0.51; 95% CI, 0.27–0.92). Malignant melanoma: 12.0 vs. 18.3 months (HR, 1.54; 95% CI, 0.67–3.43).
[99]	168 patients with advanced solid tumors treated with nivolumab from March 2009 to March 2016.	⮚ Median OS of all patients: 1.41 years (95% CI, 0.71–2.10). ⮚ Longer OS in ir thyroid disorders group vs. no ir thyroid disorders group (HR, 0.52; 95% CI, 0.25–1.11; *p* = 0.09).
[101]	51 patients with advanced NSCLC treated with pembrolizumab at Memorial Sloan Kettering Cancer Center as part of KEYNOTE-001 (NCT01295827).	⮚ Incidence of ir thyroid disorders: 21%. ⮚ Anti-thyroid antibodies positivity in ir thyroid disorders group vs. euthyroid group: (8/10 (80%) vs. 3/38(8%), *p* < 0.0001). ⮚ Median time of onset of ir thyroid disorders: 42 days. ⮚ Transient hyperthyroidism preceding hypothyroidism: 6/10 patients. ⮚ Persistent hyperthyroidism: no patient. ⮚ Most cases of hyperthyroidism and hypothyroidism: asymptomatic. ⮚ Significantly longer OS in ir thyroid disorders group compared to euthyroid group (HR, 0.29; 95% CI, 0.09–0.94; *p* = 0.04).
[121]	174 patients treated with nivolumab or pembrolizumab for metastatic or unresectable advanced cancers from September 2014 to July 2018.	⮚ Incidence of ir thyroid disorders: 16.7% (25/150 patients). ⮚ 9 patients: initial transient asymptomatic hyperthyroidism, 5 of whom developed hypothyroidism, whereas the rest were rendered euthyroid. ⮚ Median onset time: Hyperthyroidism: 10 weeks (range 4–15) Hypothyroidism: 17.8 weeks (range 5–56). ⮚ Positivity of anti-thyroid antibodies at baseline in ir thyroid disorders group vs. euthyroid group: 13/22 vs. 18/100, *p* = 0.0002. ⮚ Ir thyroid disorders group vs. euthyroid group: Median PFS: 66 vs. 27 weeks (HR, 0.50; 95% CI, 0.26–0.89, *p* = 0.02). Median OS: 156 vs. 59 weeks (HR, 0.34; 95% CI, 0.13–0.75, *p* = 0.01). ⮚ Multivariable analysis indicated ir thyroid disorders as independent prognostic factor for OS (HR, 0.42; 95% CI, 0.16–0.97, *p* = 0.04).
[122]	58 patients with metastatic NSCLC (stage IV) treated with nivolumab or pembrolizumab from January 2014 to December 2016.	⮚ Incidence of ir thyroid disorders: 32.7% (19/58 patients). ⮚ Median onset time of ir thyroid disorders: 40.0 days (IQR, 28.0–61.0). ⮚ Initial subclinical or overt hypothyroidism: 10 patients (52.6%). ⮚ Persistent hypothyroidism in all patients except 1 who developed iatrogenic subclinical thyrotoxicosis. ⮚ Initial subclinical or overt thyrotoxicosis: 9 patients (47.3%). ⮚ Persistent thyrotoxicosis in 3 patients; progression to hypothyroidism in 6 patients. ⮚ Ir thyroid disorders group vs. euthyroid group: Median OS: significantly longer in ir thyroid groups (*p* = 0.025). PFS: 118.0 (IQR, 73.0–267.0) vs. 61.0 days (IQR, 28.0–130.0; log-rank *p* = 0.014). Control rate: 15.8% vs. 0.0%, *p* = 0.011. ORR: 31.6% vs. 10.3%, *p* = 0.044. ⮚ Ir thyroid disorders predicted independently a favorable outcome: adjusted HR, 0.11; (95% CI, 0.01–0.92) for overall death; adjusted HR, 0.38; (95% CI, 0.17–0.85) for disease progression]. ⮚ Ir thyroid disorders severity was associated with durable control rate (*p* for trend = 0.008). ⮚ Status of elevated TSH ≥10.00 mU/L with or without low fT4 (overt hypothyroidism) compared to euthyroidism: Less disease progression [Log-rank *p* = 0.054, adjusted HR, 0.37 (95% CI, 0.14–0.98); *p* = 0.046]. Higher durable control rate (16.7% vs. 2.2%, *p* = 0.044). Longer median OS [Log-rank *p* = 0.152, adjusted HR 0.22 (95% CI, 0.03–1.76), *p* = 0.158]. No association of ir overt hypothyroidism with ORR (25.0% vs. 15.2%, *p* = 0.424).
[123]	105 patients treated with nivolumab for NSCLC between May 2015 and December 2016.	⮚ Ir thyroid disorders incidence: 14.3% ⮚ Ir thyroid disorders group vs. “control” group: Females: 53.3% vs. 27.8%; *p* = 0.07). Median age: 56 years vs. 62 years; *p* = 0.02). ⮚ Thyrotoxicosis (median onset: 8 weeks): 13 patients of whom 5 patients developed eventually hypothyroidism. ⮚ Isolated hypothyroidism: 2 patients (median onset time: 30 weeks). ⮚ Anti-TPOAbs positivity: 3 patients. ⮚ Transient nivolumab discontinuation: 3 patients. ⮚ Median follow-up of 9 months [95% CI, 7.5–10.3): death in the ir thyroid disorders group vs. control group: 6.7% vs. 33.3%, with a trend toward higher OS in the ir thyroid disorders group (HR, 0.16; 95% CI, 0.02–1.15; *p* = 0.07).

Abbreviations: Anti-TPOAbs, anti-thyroid peroxidase antibodies; CI, confidence interval, FDG-PET, fluorodeoxyglucose (FDG)-positron emission tomography (PET), HR, hazard ratio; IQR, interquartile range; NSCLC, non-small-cell lung cancer; OR odds ratio; ORR, objective response rate; OS, overall survival; PFS, progression-free survival; Ref, reference; vs., versus.

**Table 6:** Representative data on ir thyroid disorders in the setting of hematological malignancies.

Ref.	Type of Study N	Type of Malignacy	Treatment	Treatment Efficacy	Thyroid Immuno- Toxicity
[164]	Single-arm trial N = 70	R/R AML	Azacitidine 75 mg/m^2^ days 1 to 7 IV or SC plus nivo 3 mg/kg IV on days 1 and 14 Q 4 to 6 weeks.	ORR: 33% CR: 22%	No
[165]	Phase 1/1 b multicenter, investigator-initiated study N = 28	Relapsed hematological cancer ^a^ 3 mo or more after allogeneic HSCT	Ipilimumab at a dose of 3 or 10 mg/Kgr BW Q 3 weeks (4 doses), with additional doses Q 12 weeks for up to 60 weeks in case of clinical benefit.	Ipilimumab led to durable responses	No
[166]	Phase II, open-label study N = 121	R/R DLBCL ineligible for auto-HSCT or after auto-HSCT failure	Nivo 3 mg/kg Q 2 weeks.	Median PFS: 1.9 mo (auto-HSCT-failed cohort) 1.4 mo (auto-HSCT-ineligible cohort) Median OS: 12.2 mo (auto-HSCT-failed cohort) 5.8 mo (auto-HSCT-ineligible cohort)	No
[167]	Open-label, phase II study N = 27	Relapsed FL	R (375 mg/m^2^ IV) on days 1, 8, 15, and 22 of cycle 1 and Pembro (200 mg IV) Q 3 weeks for up to 16 cycles starting on day 2 of cycle 1.	Pre-planned interim analysis (N = 15): ORR: 80% CR: 60%	No
[142]	Single-arm phase II Study (KEYNOTE-087) N = 210	R/R cHL	Pembro 200 mg once every 3 weeks.	ORR ((95% CI): 69.0% (62.3% to 75.2%), CR: 22.4% (16.9% to 28.6%)	Hypo-thyroidism was the most common irAE with an incidence of 12.4%
[145]	Multicenter, non- comparative, phase II trial (CheckMate 205) N = 51	R/R cHL	Nivo 240 mg IV for 4 doses, followed by 12 doses of N-AVD; all doses Q 2 weeks.	Objective response rate (95% CI): 84% (71% to 93%) CR: 67% (52% to 79%) 9-mo modified PFS: 92%.	Hypo-thyroidism was the most common irAE with an incidence of 16% (No severe case)
[168]	Phase 1 study N = 23	R/R HL after auto-HSCT, or brentuximab vedotin	Nivo (at a dose of 3 mg/kgr/BW) Q 2 weeks.	Objective response rate: 87% CR: 17% PR: 70% Stable disease: 13% PFS (24 weeks): 86%	Hypo- thyroidism: 9% (Incidence)

^a^ Leukemia, lymphoma, multiple myeloma, or a neoplasm with myelodysplastic or myeloproliferative features. Abbreviations: AML, acute myeloid leukemia; auto, autologous; cHL, classic Hodgkin’s lymphoma; CR, complete response; DLBCL, diffuse large B-cell lymphoma; FL, follicular lymphoma; HL, Hodgkin’s lymphoma; HSCT, hematopoietic stem-cell transplantation; irAE, immune-related adverse event; IV, intravenously; kgr/BW, kilogram of body weight; mo, months; N, number of patients; N-AVD, nivolumab plus doxorubicin, vinblastine, and dacarbazine; nivo, nivolumab; ORR, overall response rate; OS, overall survival; Pembro, pembrolizumab; PFS, progression-free survival; PR, partial response; Q, every; R, rituximab; Ref., reference; R/R, relapsed/refractory; SC, subcutaneously.
